# Supplementing a Clay Mineral-Based Feed Additive Modulated Fecal Microbiota Composition, Liver Health, and Lipid Serum Metabolome in Dairy Cows Fed Starch-Rich Diets

**DOI:** 10.3389/fvets.2021.714545

**Published:** 2021-10-13

**Authors:** Cátia Pacífico, Thomas Hartinger, Alexander Stauder, Heidi Elisabeth Schwartz-Zimmermann, Nicole Reisinger, Johannes Faas, Qendrim Zebeli

**Affiliations:** ^1^Christian Doppler Laboratory for Innovative Gut Health Concepts of Livestock, Department for Farm Animals and Veterinary Public Health, Institute of Animal Nutrition and Functional Plant Compounds, University of Veterinary Medicine, Vienna, Austria; ^2^Christian Doppler Laboratory for Innovative Gut Health Concepts of Livestock, Department of Agrobiotechnology (IFA-Tulln), Institute of Bioanalytics and Agro-Metabolomics, University of Natural Resources and Life Sciences, Vienna, Austria; ^3^BIOMIN Research Center, BIOMIN Holding GmbH, Tulln, Austria

**Keywords:** feed additive, parity, dairy cattle, hindgut microbiota, systemic health

## Abstract

Starch-rich diets are a commonly adopted strategy in order to sustain high milk yields in dairy cows. However, these diets are known to increase the risk of gut dysbiosis and related systemic health disorders. This study aimed to evaluate the effects of supplementing a clay mineral-based feed additive (CM; Mycofix® Plus, BIOMIN) on fecal microbiota structure, fecal short-chain fatty acid (SCFA) fermentation, serum metabolome, and liver health in primiparous (PP, *n* = 8) and multiparous (MP, *n* = 16) early-lactation Simmental cows (737 ± 90 kg of live body weight). Cows were randomly assigned to either a control or CM group (55 g per cow and day) and transitioned from a diet moderate in starch (26.3 ± 1.0%) to a high starch diet (32.0 ± 0.8%). Supplementation of CM reversed the decrease in bacterial diversity, richness, and evenness (*p* < 0.05) during high-starch diet, demonstrating that CM supplementation efficiently eased hindgut dysbiosis. The CM treatment reduced levels of *Lactobacillus* in PP cows during starch-rich feeding and elevated fecal pH, indicating a healthier hindgut milieu compared with that in control. Butyrate and propionate levels were modulated by CM supplementation, with butyrate being lower in CM-treated MP cows, whereas propionate was lower in MP but higher in PP cows. Supplementing CM during high-starch feeding increased the concentrations of the main primary bile salts and secondary bile acids in the serum and improved liver function in cows as indicated by reduced levels of glutamate dehydrogenase and γ-glutamyl-transferase, as well as higher serum albumin and triglyceride concentrations. These changes and those related to lipid serum metabolome were more pronounced in PP cows as also corroborated by relevance network analysis.

**Graphical Abstract:**
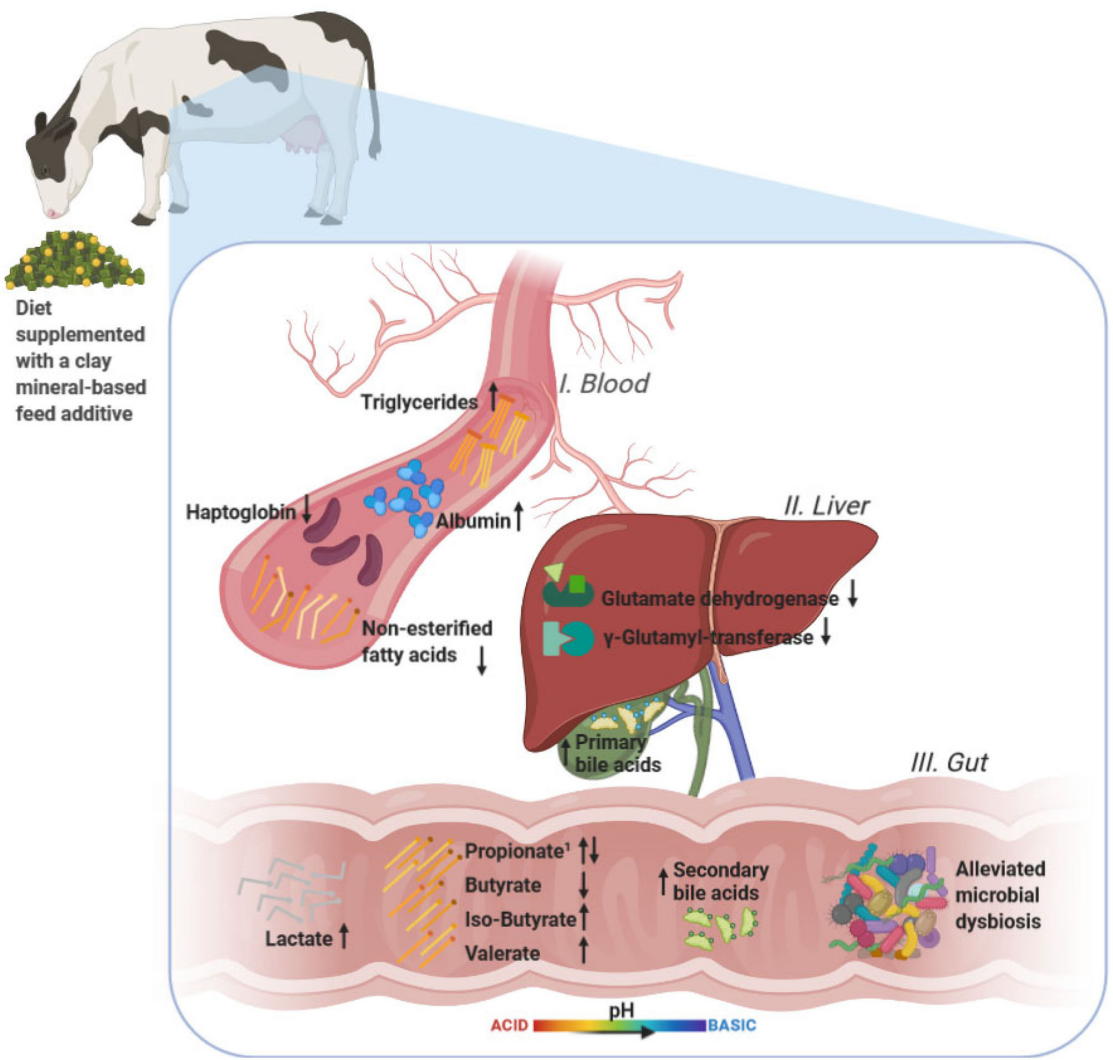
The graphical abstract provides an overview of the effects by the clay mineral-based additive on all parameters investigated in our study, i.e., bacterial community composition and milieu in the hindgut, blood, and liver health parameters, and serum metabolome. The effects are illustrated without consideration of parity or feeding phase. Created with BioRender.com. ^1^Fecal propionate concentration increased in primiparous and decreased in multiparous cows.

## Introduction

High-producing dairy cows have increased energy requirements associated with milk production. These elevated requirements are commonly met by feeding grain-rich diets typically high in starch and low in forage. However, high-grain diets can promote digestive disorders, inducing microbial dysbiosis and leaky gut syndrome in dairy cows (1). The gut and its microbiota are key modulators of animal health, and the importance of the gut–liver axis for the health of ruminants has been recently described (1). The risk of health disorders deriving from impaired liver function is especially high during early lactation (2), and the disorders induced by high-grain diets further compromise liver function by enhancing the release and translocation of microbial endotoxins into the systemic circulation (1) as well as impairing the hepatic lipid and bile acid metabolism (3). In fact, recent research showed major metabolic shifts in bile acid metabolism in response to high-starch diets (4), and this finding was consistent with increased liver enzyme concentrations, e.g., aspartate aminotransferase (AST), glutamate dehydrogenase (GLDH), γ-glutamyl-transferase (GGT), and alkaline phosphatase (AP) during early lactation (5). These effects were particularly pronounced in primiparous cows, thus highlighting the already suspected role of parity in the ability of cows to cope with starch-rich diets (6). These metabolic derailments substantially impair animal health and welfare, decrease productivity, and constitute important economic losses for the dairy industry (7).

Clay minerals, especially bentonites, possess a high adsorptive capacity and are known for their potential to bind pathogenic microorganisms and toxins (8) associated with perturbed rumen metabolism during high-grain feeding. Bentonites are known feed supplements (8), consisting mostly of montmorillonite with OH-groups on the edge sites, which can effectively buffer pH (9). Recent findings by Humer et al. (10) suggest improved liver health of non-lactating cows that were fed high-starch diets supplemented with a clay mineral-based feed additive. Interestingly, these positive effects seemed to be at least in part caused by improved rumen health, as the supplementation with the clay mineral-based feed additive supplementation lowered ruminal concentrations of lactate and biogenic amines. The same clay mineral-based additive decreased the abundance of lactate-producing or opportunistic pathogens and increased bacteria characteristic of a normobiotic rumen during high-starch feeding (11).

The current knowledge about the effects of clay mineral feed additives in ruminants derives from studies with non-lactating cows. The early lactation, however, represents a highly challenging period for dairy cows with severe metabolic stress, which is particularly true for PP animals (5), and the impact of clay mineral-based products during this lactation phase needs to be evaluated in detail. Besides, feeding a high-grain diet can increase the flow of undigested feed from the foregut to the hindgut and may likely lead to excessive fermentation, increasing the acidity of the hindgut milieu (12), which may provoke substantial shifts in the microbiota structure (13), i.e., resulting in hindgut dysbiosis and acidosis (14). Due to beneficial effects observed in previous studies, we hypothesized that supplementing clay minerals during high-grain feeding will alleviate the detrimental impact of high-starch supply on the hindgut microbiota and fermentation, preventing dysbiotic conditions in the hindgut during early lactation. For that reason, we also hypothesized that feed additive supplementation will enhance liver health and function, in both PP and MP dairy cows. Apart from bentonite, the feed additive tested in this study, as well as Humer et al. (10) and Neubauer et al. (11), also contained a mixture of plant extracts, including milk thistle. Milk thistle has been described to shape the gut microbiota beneficially (15) and exert hepatoprotective effects (16) and thus was further assumed to contribute to an improved gut and liver health status. Therefore, our study aimed to assess the impact of supplementation of CM on hindgut milieu and microbiota composition, liver health, and function as well as serum metabolome during high-grain feeding of cows at early lactation and evaluate parity-specific effects.

## Materials and Methods

### Animals, Experimental Design, and Diet

This manuscript is part of a larger study conducted at the dairy research farm of Vetmeduni Vienna (Pottenstein, Austria). Details concerning cows, experimental diets, feeding, and feed sampling, as well as feed intake data, milk yield, rumen pH, and chewing behavior in regard to the dietary starch level and parity, are reported in detail by Stauder et al. (5). Briefly, 8 PP and 16 MP lactating Simmental cows (50 ± 22 days in milk for all cows and lactation number of MP cows 4.1 ± 1.9; mean ± SD) were housed in a freestall barn equipped with 12 deep litter cubicles (2.6 × 1.25 m, straw litter) and a deep-bedded pack area (10 × 8 m) with *ad libitum* access to water and a salt block. The study started with an adaptation to the experimental barn area and training to access the individual feeding troughs for ~1 week. During the first 2 weeks, cows were fed a total mixed ration (TMR) with 60% forage and 40% concentrate (26.3% starch in the total diet, on a dry matter (DM) basis), which was considered marginal in forage and moderate in starch (M diet). Cows were assigned either to a control group without feed additive (CON) or an experimental group supplemented with a clay mineral-based product consisting of bentonite and a mixture of plant extracts (CM) such as milk thistle (Mycofix® Plus, BIOMIN Holding GmbH, Getzersdorf, Austria) starting in week 2 of feeding. Week 1 was used as baseline period. In the 3rd week, the cows were switched to a TMR considered low in forage and high in starch (H diet), containing 40% forage and 60% concentrate (32.0% starch on a DM basis) for other 4 weeks. TMRs fed during the complete experiment contained forage (50% grass silage and 50% corn silage, on a DM basis) and pelleted concentrate that contained CM in a concentration of 0.4% (on a DM basis; Table 1). Three of these 6 weeks-lasting experimental runs were conducted. Fresh TMR was offered two times a day at 07:30 and 14:30 h in equal proportions. Before the morning feeding, troughs were emptied and cleaned thoroughly. The TMR was prepared once daily with an automatic feeding system (Trioliet Triomatic T15, Oldenzaal, Netherlands) and offered *ad libitum* to ensure more than 10% feed refusals (actual orts averaged 28.1 ± 1.36% of the offered feed DM). The targeted dosage of CM was 55 g per cow and day (2.4 g per kg DM TMR), which was chosen based on previous research (10).

**Table 1:** Ingredients of the concentrate mixtures for the control cows (CON) and the clay mineral-based feed additive-supplemented cows (CM).

**Ingredients of the concentrate mixtures (%)**	**CON**	**CM**
Barley	63.0	63.2
Soybean meal	15.0	15.0
Rapeseed meal	8.0	7.4
Corn	9.0	9.0
Mineral premix^a^	2.0	2.0
Calcium carbonate	1.2	1.2
Salt	0.3	0.3
Monocalcium phosphate	0.5	0.5
Molasses	1.0	1.0
Clay mineral-based product^b^	0.0	0.4^c^

a *The mineral–vitamin premix contained 13.5% calcium, 9% magnesium, 5% phosphorus, 1.5% sodium, 1,800,000 IU vitamin A/kg, 300,00 IU vitamin D/kg, 7,500 mg vitamin E/kg, 70 mg vitamin B1/kg, 180 mg vitamin B2/kg, 145 mg vitamin B6/kg, 1,800 μg vitamin B12/kg, 1,800 mg niacin/kg, 305 mg pantothenic acid/kg, 36 mg folate/kg, 11,800 mg choline/kg, 13,500 mg manganese(II) oxide/kg, 19,800 mg zinc oxide/kg, 4,500 mg copper(II) sulfate/kg, 450 mg iodine (calcium iodine)/kg, 120 mg selene (sodium selenide)/kg, and 195 mg cobalt(II) carbonate/kg*.

b *Mycofix® Plus, BIOMIN Holding GmbH, Getzersdorf, Austria*.

c *The daily dosage per animal was 55 g*.

### Fecal Sampling and Hindgut Fermentation Profile

For the evaluation of hindgut fermentation profile and the microbiota composition, samples were collected rectally from each cow using a new palpation sleeve for each collection. Samples for fermentation pattern analysis were collected at M-wk2 (day 14), H-wk1 (day 21), H-wk2 (day 28), H-wk3 (day 35), and H-wk4 (day 42), while samples for microbiota evaluation were additionally collected at M-wk2 (day 14), H-wk1 (day 21), and H-wk4 (day 42). After collection, fecal pH was measured using a portable pH meter (Mettler-Toledo AG Analytical, Schwerzenbach, Switzerland) by direct insertion of the pH sensor into the sample. The pH meter was calibrated with pH buffers of 4.0 and 7.0. All samples were immediately frozen at −20 and −80°C until later analysis of fecal short-chain fatty acid (SCFA) and microbial community composition, respectively. Samples for SCFA and lactate analysis were thawed at room temperature for 30 min and mixed thoroughly. Subsequently, to 250 mg of fecal sample, 40 μl of a 200-ppm solution of the deuterated SCFA was added. After 5 min, 1.6 ml of HCl 0.5 M was added, and the sample was vortexed for 5 min. The samples were then centrifuged at 20,000 rcf for 5 min, and the supernatant was transferred to a 2-ml Eppendorf tube and stored at 4°C. An aliquot of 500 μl of the acidic extracts was then mixed with 1 ml of diethyl ether and vortexed for 15 min. After 5 min of centrifugation at 20,000 rcf, an aliquot of 400 μl of the etheric extract was transferred in a glass insert and dried with 25 mg of anhydrous MgSO_4_. An aliquot of 30 μl of the dried extract was then derivatized with 30 μl of *N*-*tert*-butyldimethylsilyl-*N*-methyltrifluoroacetamide with 1% *tert*-butyldimethylchlorosilane for 1 h at 60°C and subsequently for another 48 h at room temperature. The derivatized samples were then analyzed by GC-MS with a Shimadzu GC 2010 gas chromatographer coupled with a Shimadzu TQ-8050 tandem mass spectrometer (Shimadzu, Kyoto, Japan). The GC analysis was performed on a J&W DB5-MS+DG column (length 30 m, id 0.25 mm, film thickness 0.25 μm + 10 m guard column). The mass spectrometer was operated in selected ion monitoring (SIM) mode with the following conditions: electron impact ionization at 70 eV, MS transfer line temperature 280°C, MS source temperature 200°C, solvent delay 4.5 min, dwell time 300 ms, and detector gain relative to tune file.

### DNA Extraction and Sequencing

Isolation and purification of microbial DNA were performed according to Bagheri Varzaneh et al. (17) and Castillo-Lopez et al. (18), with minor modifications, using the DNeasy PowerSoil Kit (Qiagen, Hilden, Germany). Briefly, ~250 mg of fecal sample was placed in bead beating tubes, and solution C1 was added and incubated at 95°C for 5 min. Samples were centrifuged, and the supernatant was collected and placed on ice. The pellet was mixed with 100 μl of 100 mg/ml lysozyme and 10 μl of 2.5 U/ml mutanolysin (Sigma-Aldrich, St. Louis, MO, USA) and incubated at 37°C for 30 min. Afterwards, 21.3 μl of 18.8 mg/ml proteinase K (Sigma-Aldrich) was added and incubated at 37°C for 1 h. Pellets were placed in a homogenizer (FastPrep-24, MP Biomedicals, Santa Ana, CA, USA) for bead beating (three cycles of 6.5 m/s during 60 s, with 45-s pause in between cycles); and supernatant was collected after centrifugation. Cell debris and PCR inhibitors were removed through several centrifugation steps using the solutions C2–C5 provided by the manufacturer. The supernatant was then transferred to new tubes, and DNA was eluted in 100 μl of C6 buffer. After isolation, total DNA quantity was measured using the Qubit Fluorometer 2.0 (Qubit dsDNA HS Assay Kit, Thermo Fisher Scientific, Vienna, Austria) according to the manufacturer's instructions. Amplicon sequencing was performed using Illumina MiSeq paired-ends sequencing technology (Microsynth AG, Balgach, Switzerland). Targeted amplification of the hypervariable regions V3–V4 of bacterial 16S rRNA gene (2 × 250 bp) was performed using the primers 341F-ill (5′-CCTACGGGNGGCWGCAG-3′) and 802R-ill (5′-GACTACHVGGGTATCTAATCC-3′). Multiplexed libraries were constructed by ligating sequencing adapters and indices onto purified PCR products using the Nextera XT Sample Preparation Kit (Illumina, Balgach, Switzerland). Primers were trimmed, and corresponding overlapping paired-end reads were stitched by Microsynth (Microsynth AG). Sequences have been submitted to the National Center for Biotechnology Information (NCBI) sequence read archive under the accession number PRJNA646052.

### Bioinformatics Analyses

A total of 3,954,400 stitched reads were processed using the software package Quantitative Insights into Microbial Ecology (QIIME2 v2020.2) (19). Read quality was inspected using FASTQC (20) for demultiplexed Illumina fastq data with the PHRED score offset of 33. Sequence data were quality filtered using the q-score-joined plugin with a minimum acceptable PHRED score of 20 (–p-min-quality 20). Denoising into amplicon sequence variants was obtained using Deblur (21). Representative sequences and feature tables were filtered in order to exclude all sub-operational taxonomic units (sOTUs) classified as mitochondria or chloroplast sequences, yielding a total of 11,371 features. All resulting filtered sOTUs were aligned with mafft (22) and used to construct a phylogeny with fasttree2 (23). Taxonomy was assigned to sOTUs using a classify-sklearn naive Bayes taxonomy classifier trained with the 341F/802R primer set against the SILVA 132 99% OTU reference sequences (24). Taxa were then collapsed at the phylum, family, and genus levels. Only sOTUs found to represent ≥0.01% of the total sequences and in at least 25% of the samples were used for statistical evaluation.

### Blood Sampling and Liver Enzymes

Details about the blood sample collection and analyses are given by Stauder et al. (5). Briefly, blood samples were collected shortly before the morning feeding from the jugular vein in M-wk1 (day 7), M-wk2 (day 14), H-wk1 (day 21), H-wk2 (day 28), H-wk3 (day 35), and H-wk4 (day 42). Blood was collected into serum evacuated tubes for serum collection (9 ml, Vacuette, Greiner Bio-One, Kremsmuenster, Austria), K3EDTA vacutainer tubes (9 ml, Vacuette, Greiner Bio-One), and sodium fluoride vacutainer tubes (6 ml, Vacuette, Greiner Bio-One) for plasma collection. Plasma tubes were stored in the fridge immediately after collection at a temperature of 4°C for 2 h, and serum tubes were allowed to clot for 2 h at room temperature. All tubes were centrifuged at 2,000 × *g* at 4°C for 15 min (Centrifuge 5804 R, Eppendorf, Hamburg, Germany). Serum and plasma were aliquoted into 2-ml tubes (Eppendorf) and stored at −20 and −80°C (depending on target parameter) for further analysis. Concentrations of AST, AP, GGT, GLDH, β-hydroxybutyrate (BHB), non-esterified fatty acids (NEFAs), bilirubin, triglycerides (TGs), albumin, cholesterol, calcium (Ca), inorganic phosphorus (iP), and magnesium (Mg) were measured in serum samples. Glucose was measured in plasma samples (sodium fluoride) using standard enzymatic colorimetric assays, and cortisol (serum sample) was measured with a DEH3388 Cortisol ELISA, 96 well kit (Demeditec Cortisol ELISA kit, Demeditec Diagnostics GmbH, Kiel, Germany). Haptoglobin and serum amyloid A concentration in serum samples were analyzed using a commercially available cow ELISA kit (Life Diagnostics Inc., West Chester, PA, USA) and a multispecies ELISA kit (Tridelta, Maynooth, Ireland), respectively. ELISA was performed according to the manufacturer's instructions. The analyses were performed on a fully automated analyzer for clinical chemistry (Cobas 6000/c501; Roche Diagnostics GmbH, Vienna, Austria). The intraassay variation for all blood chemistry assays was ≤ 5%. Samples were analyzed in duplicate.

### Serum Metabolome

Details concerning the determination of the serum metabolome and the results related to feeding and parity are given in Pacífico et al. (4). Briefly, 10 μl of aliquots of serum samples were processed using a targeted metabolomics approach based on the Biocrates MxP® Quant 500 kit (Biocrates Life Sciences AG, Innsbruck, Austria). Analysis of serum metabolome as well as of reference standards and quality controls (provided by the manufacturer) was carried out by ultra-high-performance liquid chromatography (uHPLC) and flow injection analysis (FIA), both coupled to tandem mass spectrometry. An Agilent 1290 series UHPLC system coupled to a 6500+ QTrap mass spectrometer equipped with an Ion-Drive Turbo V® ESI source (both Sciex, Foster City, CA, USA) was used for the analysis. Chromatographic and mass spectrometric parameters were set according to manufacturer's instructions. Data analysis was carried out in Analyst 1.6.3 (Sciex) for LC-MS/MS data and in the Biocrates MetIDQ software for FIA-MS/MS data.

### Statistical Analyses

For all data, normal distribution was investigated using the Shapiro–Wilk test (*p* < 0.05). Non-normal microbial taxa were log-transformed. Statistical analyses were performed by ANOVA using the MIXED procedure of SAS (version 9.4, SAS Institute Inc., Cary, NC, USA). The model used for analyzing all data included the fixed effects of treatment (CON and CM), parity (PP and MP), and feeding phase (M-wk2, H-wk1, and H-wk4 for microbiota composition and metabolome data; and M-wk1, M-wk2, H-wk1, H-wk2, H-wk3, and H-wk4 for blood parameters, liver health, and fecal milieu parameters) as well as two-way interactions. In all analyses, the individual cows and the experimental run were considered as random effects. Baseline measurements taken before the supplementation were used as covariate in the analysis. Cow was considered as the experimental unit, and measurements obtained for the same animal but at different weeks were considered as repeated measurements. Means were compared within phase and parity using the pdiff option in the LSMEANS statement and presented as least squares means. The largest standard error of the mean is reported. Degrees of freedom were estimated with the method of Kenward–Roger. Differences were considered significant at *p* ≤ 0.05, and trends were discussed at 0.05 < *p* ≤ 0.10.

For beta-diversity, permutational multivariate ANOVA (PERMANOVA) using the *adonis* function was performed in QIIME2 v2020.2 (19). Relevance network analysis was performed using sparse partial least squares-discriminant analysis (sPLS-DA) by means of the package “mixOmics” (version 6.12.2) (25) in R studio (version 4.0.2) (RStudio Team). Associations between serum metabolome and liver health parameters, and fecal bacteria and fermentation profile were obtained via the function *network*. Figures were constructed using QIIME2 v2020.2 (19) and the R packages ggplot2 (v3.3.3) (26) and ampvis2 (v2.7.4) (27) in the R version 3.6.2.

## Results

### Hindgut Fermentation Profile

**Figure 1:**
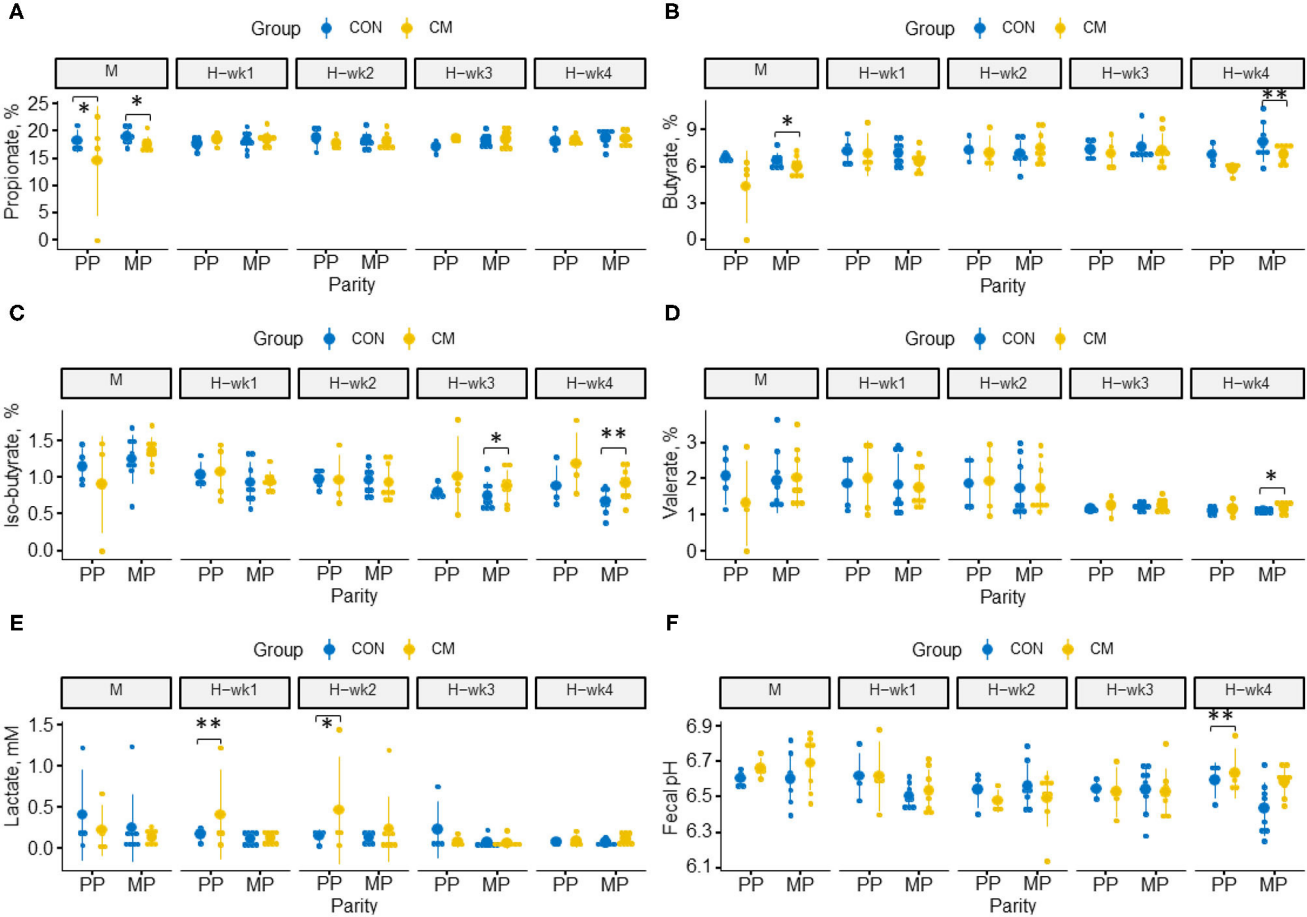
Effects of clay mineral (CM) × parity interaction on hindgut milieu parameters during moderate-grain feeding (M), followed by 4 weeks of high-grain feeding (H-wk1 to H-wk4). Dot plots illustrate fecal concentrations of **(A)** propionate, **(B)** butyrate, **(C)** isobutyrate, **(D)** valerate, **(E)** lactate, and **(F)** fecal pH. Control (CON) and treatment (CM) samples are show in blue and yellow, respectively. (*) indicates a difference by trend within parities (0.05 < *p* ≤ 0.10), and (**) indicates significant differences within parities (*p* ≤ 0.05).

The effects of CM supplementation as well as its interactions with parity or feeding phase on hindgut fermentation characteristics are presented in Figure 1. In the last week of high-starch feeding, CM-supplemented cows tended to have a higher fecal pH than cows offered the control diet (*p* = 0.07), whereas fecal lactate concentration tended to be higher during the 1st (*p* = 0.06) and 2nd weeks (*p* = 0.08) of high-starch feeding in those cows.

Isobutyrate increased during the last week of high-grain feeding in CM-treated MP cows (*p* = 0.02), and a similar trend has been observed for valerate (*p* = 0.09: Supplementary Table 1). In contrast, butyrate concentration decreased in the hindgut of CM-supplemented MP cows (*p* = 0.04), whereas no alterations were observed in PP cows. During moderate-grain feeding, propionate tended to be higher in CM-supplemented PP cows (*p* = 0.09) but lower in CM-supplemented MP cows (*p* = 0.07). As observed for the SCFA profile, lactate concentrations were affected by CM × parity (Figures 1E,F) with higher lactate during the 1st week of high-grain feeding in CM-treated PP cows (*p* = 0.03) as well as a trend for higher concentrations of lactate (*p* = 0.08) during the 2nd week of high-grain feeding.

### Effects on Fecal Bacterial Richness, Diversity, and Evenness

**Figure 2:**
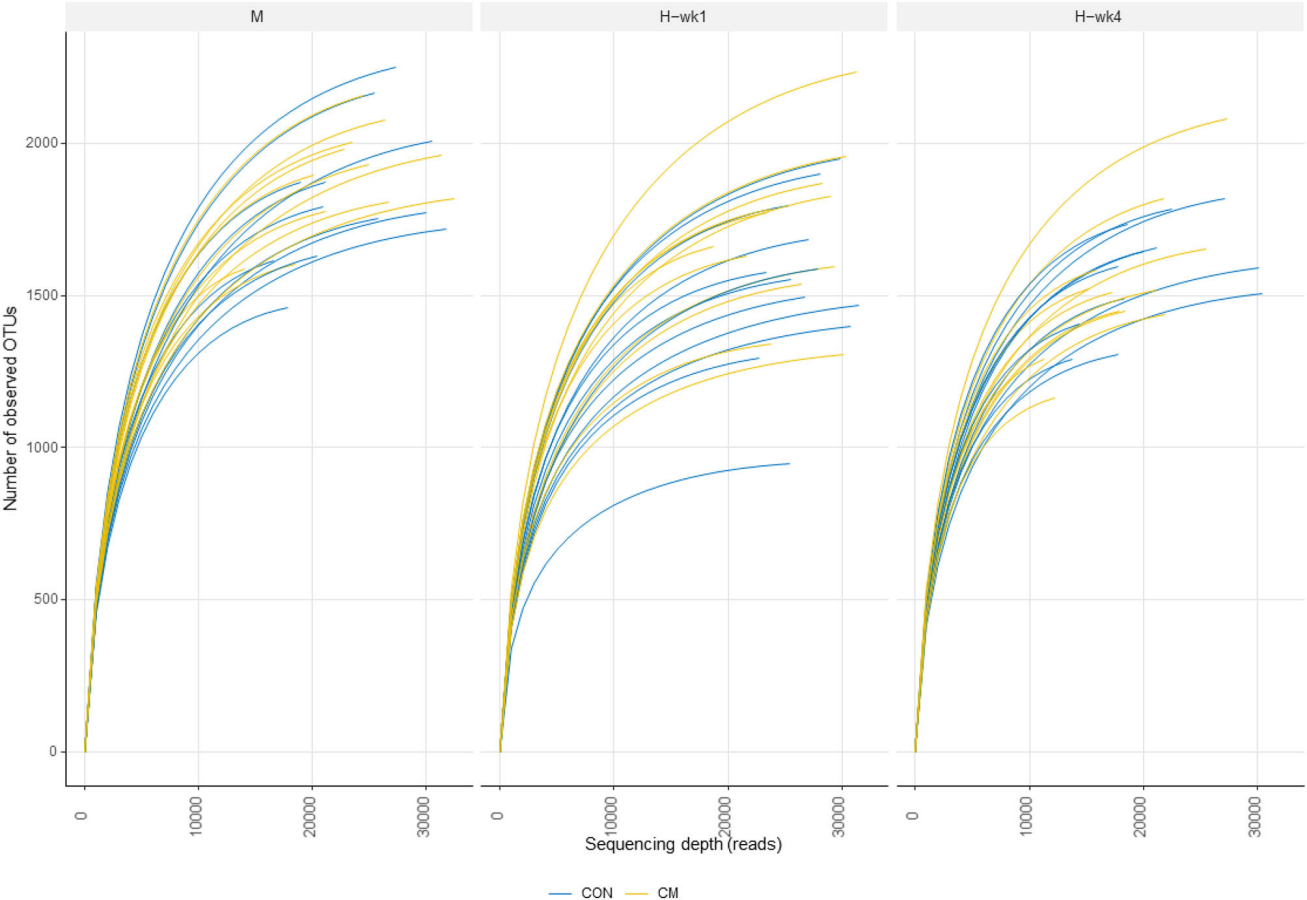
Rarefaction curves indicating the number of operational taxonomic units (OTUs) during moderate-grain feeding (M), week 1 of high-grain feeding (H-wk1), and week 4 of high-grain feeding (H-wk4). Control (CON) and treatment (CM) samples are show in blue and yellow, respectively.

**Figure 3:**
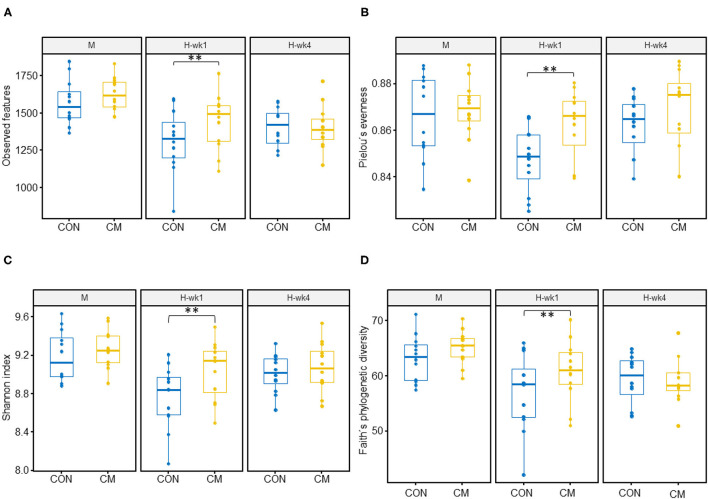
Alpha diversity indices. **(A)** Observed features, **(B)** Pielou's evenness index, **(C)** Shannon index, and **(D)** Faith's phylogenetic diversity of the fecal microbiota of early-lactation multiparous (MP) and primiparous (PP) cows during transition from moderate- to high-grain diets (after 1 and 4 weeks). (**) indicates differences between CON and CM in the same week (*p* ≤ 0.05). Control (CON) and treatment (CM) samples are show in blue and yellow, respectively.

Alpha- and beta-diversity metrics were estimated after samples were rarefied to a minimum library size of 11,260 (Figure 2) in order to account for variation in the number of sequences obtained per sample. All samples had a Good's coverage index above 0.95, indicating that the rarefaction level chosen was adequate to represent the microbial community. These metrics showed decreased diversity and evenness indices in the feces of cows when they were switched from the moderate- to high-starch diet (Figure 3). Shannon (*p* = 0.05), Simpson (*p* = 0.03), and Pielou (*p* = 0.03) indices increased in the CM group compared with the CON group. Differences between CON and the CM treatment were found particularly during 1st week of high-grain feeding. Simpson index tended to show an interaction between treatment and feeding phase (*p* = 0.09) and between treatment and parity (*p* = 0.06).

**Figure 4:**
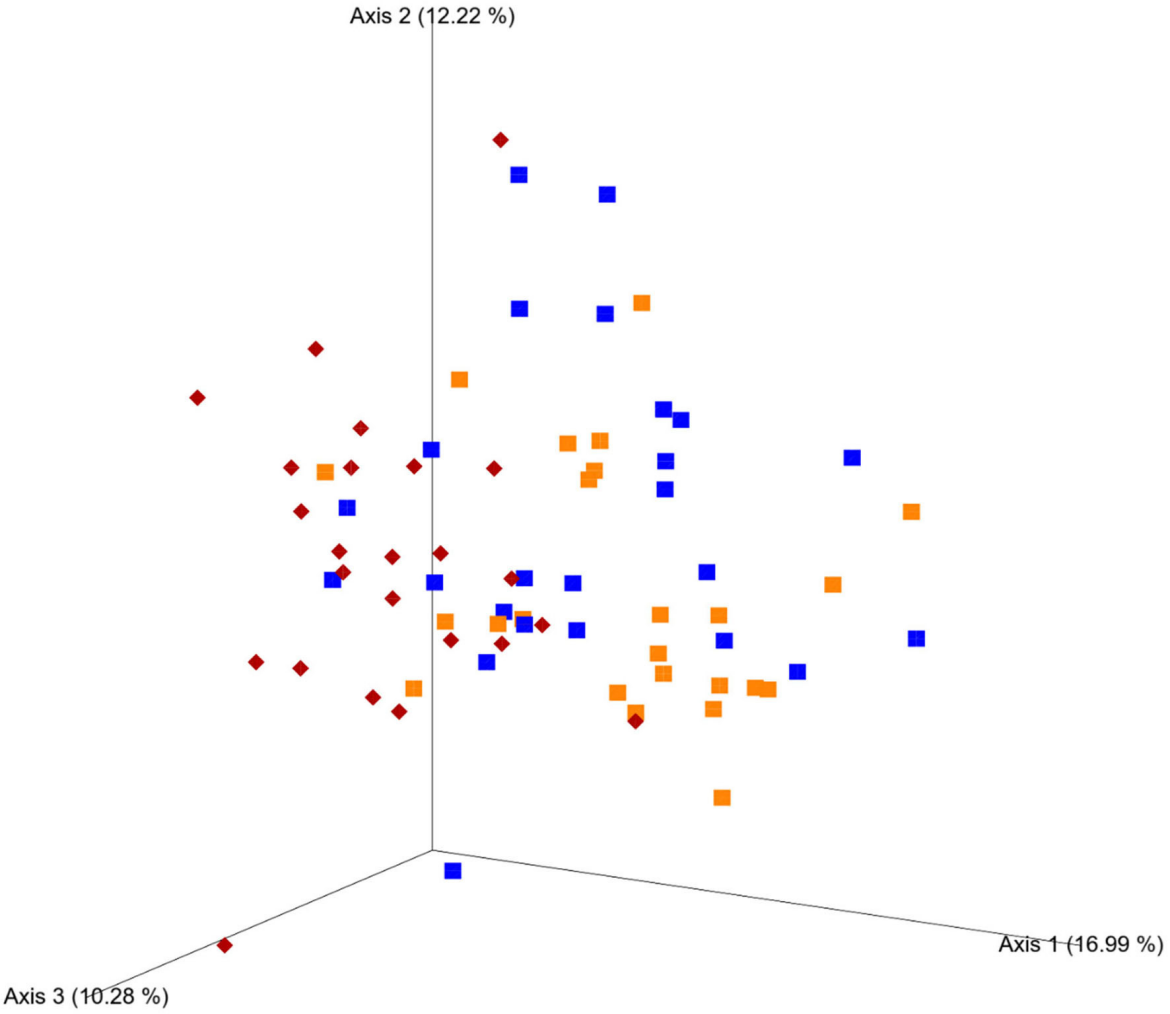
Phylogenetic clustering of fecal microbiota based on principal coordinates analysis (PCoA) plots in regard to the weighted UniFrac distance matrix. Cows fed a 40% concentrate diet (M diet, red) followed by 4 weeks of a 60% concentrate diet (H diet week 1, blue; week 4, orange). Principal components (axis) 1, 2, and 3 indicate the % of variation explained between the samples.

Principal coordinates analysis plots based on weighted UniFrac analysis show clustering of microbial communities mainly by diet, with both weeks of high-grain clustering together (Figure 4), and PERMANOVA identified starch level in the diet as the strongest effect in all distance matrices (*p* < 0.01). In the case of unweighted UniFrac and Bray–Curtis distance matrix, parity seems to play a role in the differences observed (*p* < 0.01). No effect on community structure was found for treatment using the Bray–Curtis (*p* = 0.19), weighted UniFrac (*p* = 0.26), and unweighted UniFrac (*p* = 0.06).

### Fecal Microbiota Composition

**Figure 5:**
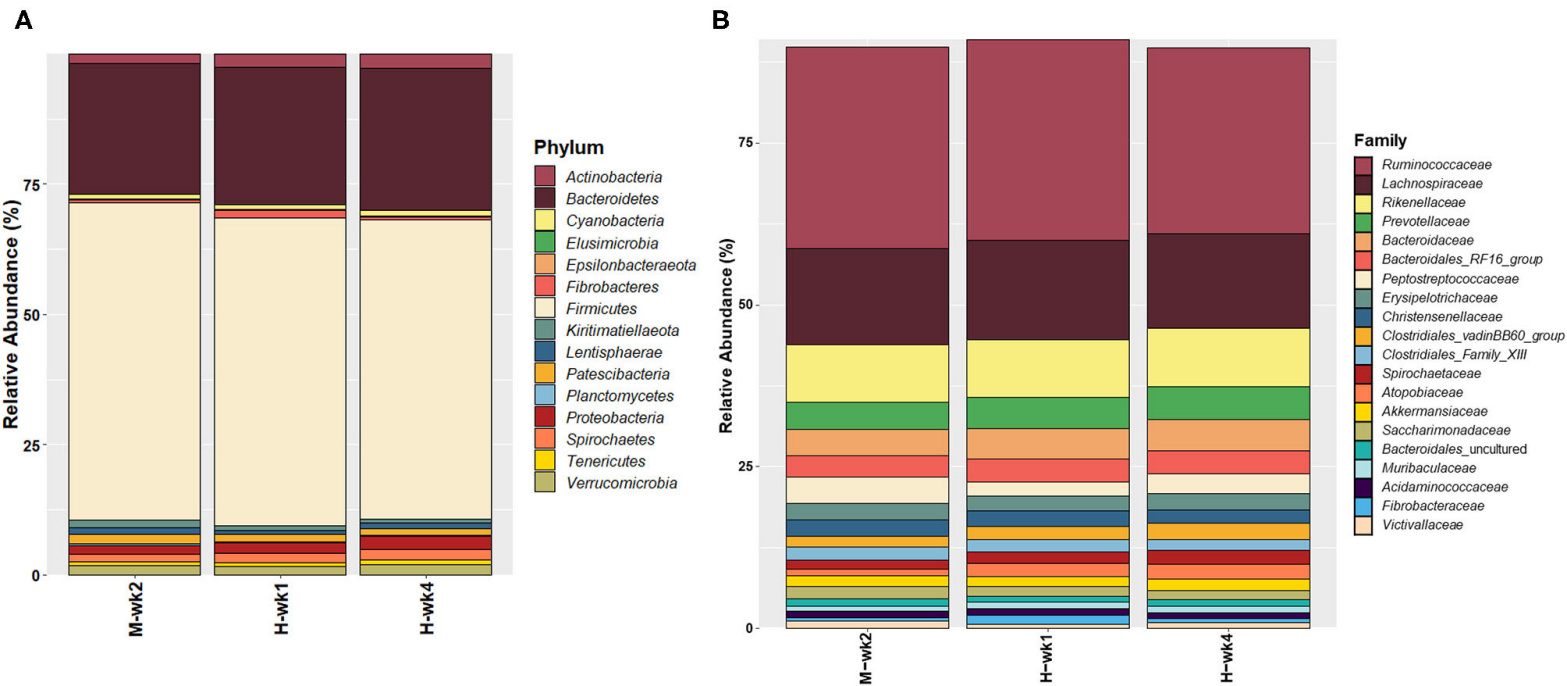
Overall microbiota composition, given by the most abundant **(A)** phyla and **(B)** families.

A total of 1,689,467 processed reads were left after feature-based filtering, ranging from 11,260 to 32,447 read counts per sample, and representing 11,371 features. The most abundant phyla can be found as summarized in Figure 5A. No significant differences were found between treatment and control at the phylum level, except Planctomycetes (*p* = 0.05) and Fibrobacteres (*p* = 0.06), which were affected by the interaction of the treatment and feeding phase. An interaction between feed additive and parity was found for Planctomycetes (*p* = 0.07), with CM-fed MP cows having a higher abundance of Planctomycetes than CON (*p* ≤ 0.1).

Approximately 99.5% of the features were assigned at the family level. The most abundant families are given on Figure 5B. Lachnospiraceae (*p* = 0.06) was more abundant in the control group, while Desulfovibrionaceae (*p* = 0.05) and Defluviitaleaceae (*p* = 0.07) were more abundant in the feces of CM-supplemented cows. Interactions between the feed additive and feeding phase were found for members of the Atopobiaceae (*p* = 0.03), Succinivibrionaceae (*p* = 0.03), Fibrobacteraceae (*p* = 0.06), Pirellulaceae (*p* = 0.05), and Bacillaceae (*p* = 0.05). The relative abundance of members of Atopobiaceae increased throughout the feeding phase and was particularly high in CON when compared with CM during the 1st week of high-grain feeding (*p* ≤ 0.05). For Succinivibrionaceae (*p* ≤ 0.05) and Pirellulaceae (*p* ≤ 1.0), differences between CON and CM were more pronounced after 4 weeks, while the low abundant Bacillaceae (*p* ≤ 1.0) was overall increased in CON during high-grain feeding. The interaction between CM and parity affected Pirellulaceae (*p* = 0.07), Marinifilaceae (*p* = 0.05), and Lactobacillaceae (*p* = 0.02). In fact, MP cows in the CON group had a higher abundance of Pirellulaceae (*p* ≤ 1.0) and Marinifilaceae (*p* ≤ 0.05). Lactobacillaceae (*p* ≤ 0.05) were more abundant in PP cows assigned to the CON group, showing a decrease in response to CM supplementation.

At the genus level, 210 bacterial genera with a relative abundance ≤ 0.01% were analyzed. Ruminococcaceae member UCG-005 (*p* = 0.01), UCG-010 (*p* = 0.05), UCG-014 (*p* = 0.08), Lachnospiraceae NK4A136 group (*p* = 0.08), *Mailhella* (*p* = 0.04), *Candidatus Soleaferrea* (*p* = 0.06), *Ruminococcus* 2 (*p* = 0.03), *Catenisphaera* (*p* = 0.08), Defluviitaleaceae UCG-011 (*p* = 0.07), *Ruminiclostridium* (*p* = 0.03), *Breznakia* (*p* = 0.08), *Tyzzerella* (*p* = 0.06), *Enterorhabdus* (*p* = 0.06), *Eisenbergiella* (*p* = 0.06), and *Sutterella* (*p* = 0.08) were affected by the feed additive supplementation (Table 2). UCG-005 and *Ruminococcus* 2 were less abundant in CM-supplemented cows, while the other Ruminococcaceae were more abundant in the CON group. *Mailhella* (*p* = 0.06), Defluviitaleaceae UCG-011 (*p* = 0.07), and *Catenisphaera* (*p* = 0.08) were overall more abundant in the CM group, while *Breznakia* (*p* = 0.08) and Lachnospiraceae NK4A136 group (*p* = 0.08) were more abundant in CON. *Tyzzerella* (*p* = 0.06) was more abundant in the feces of CM-supplemented animals. *Enterorhabdus* (*p* = 0.06), *Eisenbergiella* (*p* = 0.06), and *Sutterella* (*p* = 0.08) were tendentially impacted by CM. *Olsenella* (*p* = 0.02), *Alloprevotella* (*p* = 0.03), *Succinivibrio* (*p* = 0.04), *Dorea* (*p* = 0.02), Lachnospiraceae UCG-001 (*p* = 0.00), and Prevotellaceae Ga6A1 group (*p* = 0.03) were affected by an interaction between CM and diet. *Succinivibrio* (*p* ≤ 1.0) was more abundant in cows assigned to the CON group after 4 weeks of exposure to high-grain feeding, while *Dorea* (*p* ≤ 0.05) was more abundant in CON when exposed to a moderate level of grain in the diet. An interaction between feed additive and parity was found for Prevotellaceae UCG-004 (*p* = 0.03), *Coprococcus* 3 (*p* = 0.04), *Ruminobacter* (*p* = 0.07), *Lactobacillus* (*p* = 0.03), family XIII UCG-001 (*p* = 0.04), *Breznakia* (*p* = 0.02), *Eisenbergiella* (*p* < 0.01), *Intestinimonas* (*p* < 0.01), and Lachnospiraceae UCG-007 (*p* = 0.07; Table 3). Prevotellaceae UCG-004, family XIII UCG-001, and *Lactobacillus* (*p* ≤ 0.05) were lower in CM-supplemented PP cows when compared with CON. The opposite was found for *Eisenbergiella* (*p* ≤ 0.05). The CM treatment increased the relative abundance of *Intestinimonas* in MP cows and decreased it in PP cows (*p* ≤ 0.05). Lachnospiraceae UCG-007 decreased in CM-fed MP cows (*p* ≤ 1.0).

**Table 2:** Relative abundance of bacterial genera significantly (*p* ≤ 0.05) or tendentially (0.05 < *p* ≤ 0.10) affected by feed additive (CM) or an interaction of feed additive with feeding phase.

**Genus**	**Additive**	**Feeding phase**			**SEM**	***p*** **-value**	
		**M-wk2**	**H-wk1**	**H-wk4**		**Additive**	**Phase × Additive**
Ruminococcaceae UCG-005	CON	9.36	11.5^y^	9.84^a^	0.47	0.01	0.42
	CM	8.88	10.3^z^	8.12^b^			
Ruminococcaceae UCG-010	CON	7.21^b^	7.37	6.75^z^	0.56	0.05	0.19
	CM	8.94^a^	7.15	8.16^y^			
Ruminococcaceae UCG-014	CON	1.91	1.57	1.86	0.26	0.08	0.82
	CM	2.11	1.75	2.26			
*Olsenella*	CON	0.36^z^	1.20^b^	2.05	0.57	0.92	0.02
	CM	1.20^y^	1.29^a^	2.35			
Prevotellaceae UCG-004	CON	1.18	1.29	1.59^a^	0.13	0.15	0.09
	CM	1.00	1.40	1.29^b^			
Prevotellaceae UCG-003	CON	1.28	1.01	1.33^a^	0.13	0.49	0.06
	CM	1.31	1.16	0.96^b^			
*Alloprevotella*	CON	0.86	1.26	0.82	0.15	0.73	0.03
	CM	0.72	0.84	1.14			
*Fibrobacter*	CON	0.25	1.30	0.33	0.38	0.46	0.06
	CM	0.82	1.32	0.78			
*Succinivibrio*	CON	0.31	0.49	1.24^y^	0.21	0.19	0.04
	CM	0.21	0.50	0.71^z^			
Lachnospiraceae NK4A136 group	CON	0.54	0.69	0.53	0.08	0.08	0.42
	CM	0.32	0.54	0.44			
*Dorea*	CON	0.56^a^	0.46	0.46	0.04	0.41	0.02
	CM	0.43^b^	0.51	0.44			
dgA-11 gut group	CON	0.44	0.42	0.32	0.05	0.97	0.07
	CM	0.46	0.35	0.38			
*Mailhella*	CON	0.33	0.28^z^	0.34	0.04	0.06	0.91
	CM	0.39	0.37^y^	0.41			
*Candidatus Soleaferrea*	CON	0.36^z^	0.32^z^	0.31	0.03	0.06	0.68
	CM	0.43^y^	0.37^y^	0.34			
*Ruminococcus* 2	CON	0.51	0.43	0.36^a^	0.12	0.03	0.57
	CM	0.27	0.28	0.21^b^			
*Catenisphaera*	CON	0.07	0.06	0.08	0.30	0.08	0.67
	CM	0.45	0.38	0.54			
Lachnospiraceae UCG-001	CON	0.22	0.12^b^	0.27^a^	0.04	0.52	0.00
	CM	0.17	0.18^a^	0.11^b^			
p-1088-a5 gut group	CON	0.24	0.12	0.16	0.06	0.68	0.06
	CM	0.32	0.16	0.05			
Ruminococcaceae UCG-004	CON	0.24	0.18	0.14	0.02	0.43	0.07
	CM	0.21	0.22	0.17			
Prevotellaceae Ga6A1 group	CON	0.16^y^	0.11^z^	0.15	0.03	0.38	0.03
	CM	0.08^z^	0.17^y^	0.09			
*Anaerosporobacter*	CON	0.17	0.05	0.15^a^	0.03	0.41	0.10
	CM	0.12	0.11	0.07^b^			
Defluviitaleaceae UCG-011	CON	0.09^z^	0.09	0.08	0.01	0.07	0.60
	CM	0.11^y^	0.11	0.09			
*Dielma*	CON	0.11	0.15^a^	0.09	0.01	0.23	0.07
	CM	0.08	0.10^b^	0.10			
*Ruminiclostridium*	CON	0.05	0.04	0.03	0.01	0.03	1.00
	CM	0.07	0.05	0.03			
*Breznakia*	CON	0.03	0.05	0.05	0.01	0.08	0.90
	CM	0.02	0.04	0.05			
*Tyzzerella*	CON	0.02^z^	0.03	0.02	0.01	0.06	0.39
	CM	0.04^y^	0.04	0.04			
*Enterorhabdus*	CON	0.03^a^	0.01	0.03	0.01	0.06	0.35
	CM	0.02^b^	0.02	0.02			
*Eisenbergiella*	CON	0.02	0.02	0.02^b^	0.00	0.06	0.26
	CM	0.02	0.03	0.02^a^			
*Sutterella*	CON	0.01	0.03	0.03	0.01	0.08	0.95
	CM	0.02	0.03	0.03			
*Mogibacterium*	CON	0.02^a^	0.00	0.01	0.00	0.51	0.07
	CM	0.01^b^	0.01	0.01			

a, b *Indicate significant differences between CON and CM in the same week (p ≤ 0.05)*.

y, z *Indicate trends between CON and CM in the same week*.

**Table 3:** Relative abundance of bacterial genera significantly (*p* ≤ 0.05) or tendentially (0.05 < *p* ≤ 0.10) affected by an interaction of feed additive (CM) with parity.

**Genus**	**Additive**	**Parity**		**SEM**	***p*-value**
		**MP**	**PP**		**Parity × Additive**
Prevotellaceae UCG-004	CON	1.18	1.53^a^	0.13	0.03
	CM	1.25	1.22^b^		
*Coprococcus* 3	CON	0.67	0.64	0.09	0.04
	CM	0.71	0.56		
*Ruminobacter*	CON	0.27	0.26	0.10	0.07
	CM	0.34	0.07		
*Lactobacillus*	CON	0.04	0.05^a^	0.03	0.03
	CM	0.04	0.02^b^		
Family XIII UCG-001	CON	0.04	0.05^a^	0.01	0.04
	CM	0.04	0.03^b^		
*Breznakia*	CON	0.04	0.05	0.01	0.02
	CM	0.04	0.03		
*Eisenbergiella*	CON	0.03	0.01^b^	0.00	0.00
	CM	0.02	0.02^a^		
*Intestinimonas*	CON	0.01^b^	0.03^a^	0.01	0.00
	CM	0.02^a^	0.01^b^		
Lachnospiraceae UCG-007	CON	0.02^y^	0.01	0.01	0.07
	CM	0.01^z^	0.02		

a, b *Indicate significant differences between CON and CM in the same week (p ≤ 0.05)*.

y, z *Indicate trends between CON and CM in the same week*.

### Blood and Liver Health Parameters

**Figure 6:**
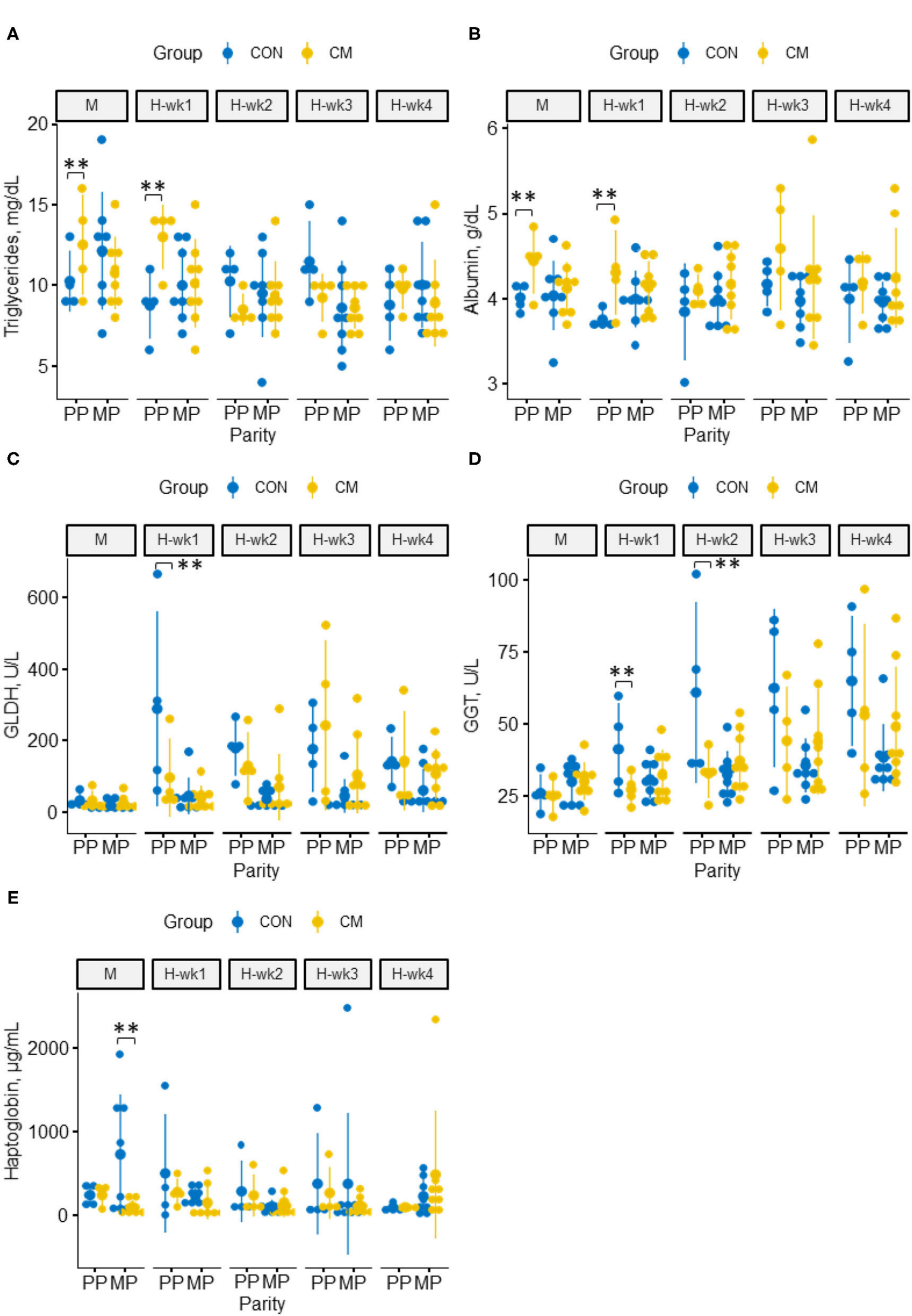
Effect of CM supplementation in primiparous and multiparous cows fed a 40% concentrate diet for 1 week (M) and a 60% concentrate diet for 4 weeks (H-wk1 to H-wk4) on blood metabolites and liver health parameters. **(A)** triglycerides, **(B)** albumin, **(C)** GLDH, **(D)** GGT, **(E)** haptoglobin. **indicates significant differences within parities (*p* ≤ 0.05).

The CM-supplemented cows showed overall less NEFA (*p* = 0.05) but higher albumin concentrations (*p* = 0.02) than the control-fed animals (Figure 6). Similarly, TGs increased in CM-supplemented cows in the 1st week of high-grain feeding (*p* = 0.05). During moderate-grain feeding and the 1st week of high-grain feeding, we observed interactions between parity and CM with higher concentrations of albumin (*p* < 0.01) and TG (*p* = 0.04) in CM-supplemented PP cows compared with PP control cows, whereas no differences were observed in MP cows. Concentrations of GLDH (*p* = 0.02) and GGT (*p* = 0.03 and 0.01 for H-wk1 and H-wk2, respectively) were as well-decreased in CM-supplemented PP cows during the first half of the high-grain challenge. The CM-treated MP cows, in turn, showed lower haptoglobin concentrations than the MP control cows during moderate-grain feeding (*p* = 0.02), whereas no difference was observed for PP cows in this feeding phase. The supplementation with CM had no effect on mineral levels in the blood (Supplementary Table 2). Similarly, concentrations of bilirubin, cholesterol, β-hydroxy-butyric acid, and serum amyloid A were not affected by CM supplementation or its interactions with parity or feeding phase.

### Effects on Lipid and Bile Metabolism

Bile acids cholic acid (CA; *p* = 0.01), chenodeoxycholic acid (CDCA) (*p* = 0.03), and deoxycholic acid (DCA) (*p* = 0.02) were all greater in serum of CM-supplemented cows than the control group (Supplementary Table 3). An interaction between feeding phase and parity was found for diglycerides DG(16:0_16:0) (*p* = 0.01) and DG(18:1_20:1) (*p* = 0.03). Sphingomyelin SM C26:1 was more abundant in CM-fed PP cows (*p* = 0.04). Phosphatidylcholine PC a C26:0 was more abundant in MP cows, particularly in the group fed with the feed additive (*p* = 0.04). Lysophosphatidylcholines lysoPC a C14:0 (*p* = 0.05) and C24:0 (*p* = 0.05) were affected by the CM supplementation. This effect was more pronounced in MP cows.

After 1 week, supplementing the CM decreased the serum levels of glutamate of PP cows (*p* < 0.01). Glycolithocolic acid was substantially increased in PP cows from the CM group but exerted no effect in MP cows. A significant interaction was found for the lysophosphatidylcholines lysoPC a 16:0 (*p* = 0.02), lysoPC a 16:1 (*p* = 0.01), and lysoPC a 18:2 (*p* = 0.02), which decreased in the CM group of MP cows. LysoPC a 18:2, however, increased in CM-fed PP cows. Several TGs were impacted by the CM supplementation. The levels of TG(16:0_35:2), TG(16:1_34:1), TG(16:1_34:2), TG(16:1_36:1), TG(18:0_34:3), TG(18:1_32:2), TG(18:1_36:5), TG(18:2_32:1), and TG(18:2_36:0) were all increased by the CM (*p* ≤ 0.05), with CM-fed cows having 18% more TG than CON. An additional interaction between CM and parity was found for TG(16:1_34:2), TG(16:1_36:1), and TG(20:1_24:3) (*p* ≤ 0.05) in PP cows in particular. Changes at the level of the phosphatidylcholines were also observed. The CM group had overall more PC ae C 30:2 (*p* = 0.02) and PC aa C30:2 (*p* = 0.03) and less PC aa C38:0 (*p* = 0.03) and PC aa C42:5 (*p* = 0.03). Cows fed with the feed additive had overall more lysoPC a C28:1 (*p* = 0.03), in particular PP animals. Triglycerides TG(14:0_36:2), TG(17:0_32:1), TG(18:1_35:2), and TG(18:2_30:1) were more abundant in the serum of CM-fed animals than CON (*p* ≤ 0.05). Additional effects between the CM and parity were found for TG(18:1_35:2; *p* = 0.01) and TG(18:2_32:2; *p* = 0.01).

### Relevance Network Analysis

**Figure 7:**
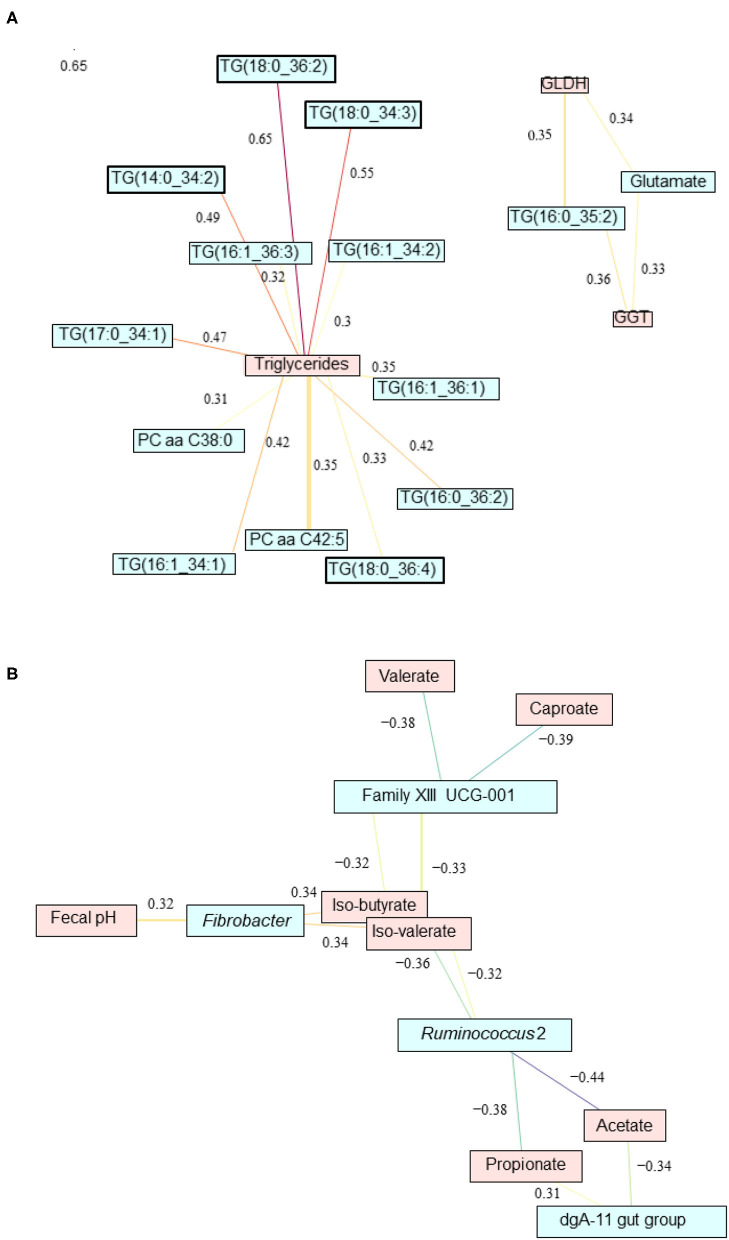
Relevance network analysis showing the most significant pairwise associations **(A)** between liver parameters and serum metabolome and **(B)** between fecal microbiota and fermentation profile impacted by feed additive feeding assessed using sparse partial least squares regression (|r| = 0.3).

Relevance network analysis was used to identify the most important serum metabolites associated with liver health and the main fecal bacteria associated with metabolic pathways impacted by CM supplementation. The most significant pairwise associations between serum metabolome and liver health indicators (|r| = 0.3) are given in Figure 7A. In particular, triglycerides TG(18:0_36:2), TG(18:0_34:3), TG(16:1_34:2), TG(16:1_36:3), TG(14:0_34:2), TG(17:0_34:1), TG(16:1_36:1), TG(16:0_36:2), TG(18:0_36:4), and TG(16:0_34:1) and phosphatidylcholines PC aa C42:5 and PC aa C38:0 were all strongly correlated with the total amount of triglycerides measured in blood. TG(16:0_35:2) and glutamate are correlated with both GGT and GLDH. Significant pairwise associations between fecal microbiota and fermentation profile (|r| = 0.3) are given in Figure 7B. *Fibrobacter* was positively correlated with fecal pH, isobutyrate, and isovalerate. Members of the family XIII UCG-001 were negatively correlated with valerate, caproate, isobutyrate, and isovalerate. *Ruminococcus* 2 were found to be negatively correlated with isobutyrate, isovalerate, acetate, and propionate. However, dga-11 gut group was positively correlated with propionate but negatively correlated to acetate.

## Discussion

Our study aimed to assess whether the supplementation of a clay mineral-based product can alleviate the detrimental impact of high-starch diets on the microbiota and fermentation pattern in the hindgut and help to maintain healthy liver function of cows during early lactation. As starch-rich diets induced severe disorders in the rumen, such as a higher subacute rumen acidosis index, as well as impaired liver health, i.e., elevated serum concentrations of GGT, GLDH, and AST, in particular in PP cows (5, 6), a second aim of this study was to investigate if this perturbed liver health and metabolism are related to gut health and whether CM supplementation will affect variables of liver health and serum metabolome in a parity-specific way. As both groups were similar in feed intake and milk performance (Supplementary Table 4), all changes observed in the hindgut and at the metabolic level can directly be attributed to CM.

The most interesting finding of the study was an improvement of the diversity of fecal microbiota in the CM group during the 1st week of exposure to a high-grain diet. This corresponds to the most challenging period when it comes to severity of rumen acidosis, particularly in PP cows (5). By comparing CM and CON groups during the 1st week cows were offered a high-grain diet, CM-supplemented cows showed higher bacterial diversity, richness, and evenness, revealing that CM clearly ameliorates the deleterious effects of high-grain feeding, which confirms our first hypothesis. High amounts of grain in the diet lead to decreased richness and diversity of the fecal microbiota community, as observed for CON cows in our study, which is caused by a substantial flow of starch to the hindgut (11). An increased bypass of fermentable starch from the rumen to the lower gastrointestinal tract is typical for subacute rumen acidosis, i.e., when the ruminal function is significantly impaired (28); it is plausible that CM improved the rumen function and so prevented an excessive nutrient inflow to the hindgut, which consequently averted the establishment of severe dysbiotic states. Similarly, the milk thistle may have contributed to this observed amelioration, as gut microbiota-modulating effects by milk thistle-derived bioactive compounds have been observed in culture media (15) and mice (29); but confirmation in ruminants is yet pending. The beneficial impact of CM on the hindgut gut microbiota was also apparent at the metabolome level, where we observed overall higher serum concentrations of primary and secondary bile salts in CM-treated cows. Since the gut microbiota regulates the bile salt pool (30), which decreases in case of impaired microbial transformation activity, higher bile salt levels in CM-treated cows again indicated an alleviated dysbiosis and consequently were associated with improved enterohepatic circulation.

As observed in alpha diversity metrics, the benefits of CM on the fecal microbiota were further shown at the genus level, as the supplementation of CM substantially decreased *Lactobacillus* in PP cows, a genus that harbors amylolytic and lactic acid-producing bacteria. Remarkably, clay minerals were previously found to reduce lactobacilli in the rumen (11), indicating a counteracting effect of the feed additive against some lactate producers along the bovine gastrointestinal tract. Due to their amylolytic metabolism, the reduced abundance of *Lactobacillus* may again be the consequence of a diminished flow of concentrate to the hindgut. Furthermore, less lactobacilli might also be a consequence of a lowered overall hindgut pH as suggested by fecal pH data. CM-treated PP cows had slightly higher fecal lactate concentrations in the 1st week of high-grain feeding, which may be ascribed to the higher abundance of *Eisenbergiella*, a genus that also comprises lactate producers (31). However, the increase in fecal lactate is deemed to be not biologically meaningful, as the concentrations are generally very low (32).

Similar to *Lactobacillus*, the reduced presence of Succinivibrionaceae in CM-supplemented cows during the last week of the experiment can be interpreted as favorable in terms of gut health, as this family comprises a variety of potential pathogens and strongly proliferates when large quantities of nutrients reach the hindgut (6, 33), which in turn could again point to an improved rumen function in CM cows. The decrease of cellulolytic Lachnospiraceae NK4A136 group and Ruminococcaceae UCG-005 observed in CM-treated cows was in line with the lower butyrate levels in CM-treated MP cows and would match the assumption of an improved rumen milieu by CM that causes less potentially fermentable fiber reaching the lower gut, where it would otherwise be utilized by these fibrolytic bacteria. Indeed, the decrease in fecal butyrate concentration of approximately 1.2 percentage units in CM-supplemented MP cows during H-wk4 was accompanied by a higher fecal pH than cows offered the control diet. Apart from butyrate, CM had only a minor impact on fecal SCFA concentrations, which corresponds to recent findings in the rumen (11) and thus implies that CM marginally influences fermentation patterns along the bovine gastrointestinal tract.

When looking at the systemic level, the results validate our second hypothesis and show that the CM supplementation appeared to enable a smoother initial adaptation of PP cows to high-grain diets as revealed by several blood parameters at the beginning of high-grain feeding. The reduced concentrations of GLDH and GGT in CM-treated PP cows during the first half of high-grain feeding indicate a less challenged liver function, which is also in line with the lower serum levels of glutamate observed for CM-treated PP cows and the pairwise associations revealed by relevant network analysis. Likewise, increased albumin levels in CM-treated PP cows also suggest an enhanced synthesis capacity of their hepatic tissue as well as a protective effect by CM against liver fat infiltration damage in those cows (34). In this context, the metabolomics data also supported the hypothesized beneficial effect of CM on the gut–liver axis, as the majority of bile acids is reabsorbed from the gut and transported back to the liver via the portal vein where they are bound to albumin and made available for secretion (35). Together with the increase in albumin, the overall higher serum concentrations of the main primary bile salts, CA and CDCA, as well as the secondary bile acid DCA are further indicators of an improved enterohepatic circulation in CM-supplemented cows. Based on these observations, CM supplementation seemed to be effective in alleviating the impacts of high-grain feeding and metabolic stress in PP cows, which confirms our hypothesis of an improved systemic health status. Indeed, when treated with CM, liver enzyme concentrations in PP cows remained at levels similar to those of MP cows, which are better coping with high-grain feeding regimens (5, 6). Likewise, the overall lower NEFA levels and higher TG concentrations during the 1st week of high-grain feeding with CM in both PP and MP cows provide further evidence for an improved hepatic esterification activity along with less TG accumulation (2, 36). Moreover, high NEFA levels impede hepatic gluconeogenesis in dairy cows (37), and the lower NEFA concentrations in CM cows may contribute to an enhanced hepatocyte function.

Changes at the level of the serum metabolome were found already during moderate-grain feeding. Interestingly, several serum TGs were significantly increased by CM supplementation during the 1st week of high-grain feeding, which is consistent with an increase in the levels of total TG toward an improved liver health. Several of these individual TGs identified in the serum metabolome may therefore be further explored as potential biomarkers for an improved liver health.

The exact mode of action of CM on liver health remains to be entirely understood. However, as already proposed in a similar way (10), it seems plausible that less harmful compounds such as endotoxins entered the systemic circulation due to adsorption by CM (8), consequently disburdening the hepatic detoxification activity (38) and enabling a focus on energy metabolism. Additionally, CM contains a mixture of plant extracts, such as milk thistle extract, which might have contributed to the beneficial impact on liver function as their bioactive ingredients are discussed for exerting hepatoprotective and beneficial health effects in ruminants (39, 40). Thereby, these hepatoprotective effects have mainly been associated with polyphenolic antioxidants and silymarin, a flavonoid mixture of diverse flavonolignans, which is highly present in milk thistle. These compounds improve animal health via different modes of action, such as decreasing the pro-inflammatory NF-κB signaling, scavenging free radicals or inhibiting their formation, and regulating antioxidant enzymes expression, e.g., upregulation of superoxide dismutase in mitochondria (16). The application of clay mineral-based feed additives was previously discussed to decrease the bioavailability of Ca and other minerals by means of cation adsorption (41). Our findings on serum magnesium, inorganic phosphorus, and calcium concentrations, however, do not support this assumption, as all mineral concentrations were within the respective physiological ranges for dairy cows (42, 43).

Overall, feeding high-grain diets during early lactation may lead to an escape of undigested feed from the rumen to the hindgut, which results in increased hindgut fermentation, shifts in the microbial community composition, and perturbed liver function, especially in PP cows. Our study provides evidence that supplementing CM, including bentonite and plant extracts, holds potential to enhance liver health and metabolism during the transition period and early lactation of dairy cows. The improvements are likely initiated by the adsorbing capacity of the clay minerals against deleterious microbial metabolites produced upon the grain-induced dysbiosis and the hepatoprotective effects of the plant extracts. The CM-related improvements in the fecal microbiota structure are linked to the amended health status and function of the liver, particularly in PP cows. This highlights, first, the importance of the gut–liver axis in starch-rich feeding in cattle and, second, cow parity as an important biological factor to consider when designing healthy feeding management strategies for cattle.

## Data Availability Statement

The datasets presented in this study can be found in online repositories. The names of the repository/repositories and accession number(s) can be found in the article/Supplementary Material.

## Ethics Statement

The animal study was reviewed and all procedures involving animal handling and treatment were approved by Institutional Ethics and Animal Welfare Committee of the University of Veterinary Medicine (Vetmeduni Vienna, Austria) and the national authority according to §26 of the Law for Animal Experiments, Tierversuchsgesetz 2012-TVG (GZ: 68.205/0023-V/3b/2018).

## Author Contributions

AS, CP, HS-Z, JF, NR, QZ, and TH: formal analysis and writing—review and editing. AS, CP, and TH: investigation and methodology. CP and TH: visualization and writing—original draft. HS-Z, NR, and QZ: funding acquisition and project administration. HS-Z, JF, NR, and QZ: resources. QZ: conceptualization and supervision. All authors contributed to the article and approved the submitted version.

## Funding

This research was funded by the Österreichische Forschungsförderungsgesellschaft (FFG, Frontrunner program line: grant number 866384), the Austrian Federal Ministry for Digital and Economic Affairs, and the National Foundation for Research, Technology and Development through the Christian Doppler Research Society. The research of TH was funded by the Deutsche Forschungsgemeinschaft (DFG, German Research Foundation), 447776988.

## Conflict of Interest

NR and JF are employed by BIOMIN Holding GmbH, a company that manufactures and trades feed additives. The remaining authors declare that the research was conducted in the absence of any commercial or financial relationships that could be construed as a potential conflict of interest.

## Publisher's Note

All claims expressed in this article are solely those of the authors and do not necessarily represent those of their affiliated organizations, or those of the publisher, the editors and the reviewers. Any product that may be evaluated in this article, or claim that may be made by its manufacturer, is not guaranteed or endorsed by the publisher.
