# Supplementary material for: Supplementing a Clay Mineral-Based Feed Additive Modulated Fecal Microbiota Composition, Liver Health, and Lipid Serum Metabolome in Dairy Cows Fed Starch-Rich Diets
Source: Front Vet Sci. 2021 Oct 13;8:714545. doi: 10.3389/fvets.2021.714545 (PMC8548638; doi:10.3389/fvets.2021.714545)
Supplement: Supplementary file 1 [file Data_Sheet_1.docx]

**TABLE S1**. Effects of clay mineral-based feed additive (CM), interaction CM × parity, and CM × phase on hindgut milieu parameters.

|  |  | Primiparous | | Multiparous | |  | P-values | | |  |
| --- | --- | --- | --- | --- | --- | --- | --- | --- | --- | --- |
| Item | Phase^1^ | CON^2^ | CM^3^ | CON | CM | SEM | CM | CM × Parity | CM × Phase | |
| Total SCFA^4^ [mM] | M | 42.9 | 39.0 | 40.0 | 35.8 | 5.35 | 0.30 | 0.98 | 0.27 | |
|  | H-wk1 | 44.4 | 44.8 | 47.0 | 46.6 | 3.33 | 0.99 | 0.88 |  | |
|  | H-wk2 | 50.8 | 48.2 | 46.7 | 49.9 | 6.96 | 0.95 | 0.60 |  | |
|  | H-wk3 | 45.6 | 45.3 | 43.8 | 48.8 | 3.67 | 0.42 | 0.37 |  | |
|  | H-wk4 | 42.0 | 41.9 | 43.1^y^ | 34.7^z^ | 5.33 | 0.33 | 0.33 |  | |
| Acetate [%] | M | 71.2 | 69.5 | 70.7^z^ | 72.6^y^ | 1.47 | 0.94 | 0.10 | 0.51 | |
|  | H-wk1 | 71.7 | 70.4 | 71.4 | 71.9 | 1.26 | 0.68 | 0.38 |  | |
|  | H-wk2 | 70.5 | 71.2 | 71.5 | 71.4 | 1.70 | 0.83 | 0.76 |  | |
|  | H-wk3 | 73.1 | 71.6 | 71.7 | 71.5 | 1.29 | 0.40 | 0.55 |  | |
|  | H-wk4 | 72.5 | 73.0 | 71.1 | 72.0 | 1.02 | 0.38 | 0.84 |  | |
| Propionate [%] | M | 18.2^z^ | 20.7^y^ | 19.0^y^ | 17.5^z^ | 1.11 | 0.55 | 0.02 | 0.45 | |
|  | H-wk1 | 17.7 | 18.4 | 18.3 | 18.6 | 0.86 | 0.43 | 0.76 |  | |
|  | H-wk2 | 18.8 | 17.8 | 18.3 | 18.1 | 0.92 | 0.42 | 0.55 |  | |
|  | H-wk3 | 17.2 | 18.7 | 18.4 | 18.6 | 0.76 | 0.15 | 0.29 |  | |
|  | H-wk4 | 18.2 | 18.4 | 18.8 | 18.6 | 0.82 | 0.99 | 0.72 |  | |
| Iso-Butyrate [%] | M | 1.15 | 1.09 | 1.25 | 1.37 | 0.19 | 0.81 | 0.52 | 0.18 | |
|  | H-wk1 | 1.04 | 0.98 | 0.93 | 1.00 | 0.15 | 0.96 | 0.60 |  | |
|  | H-wk2 | 0.97 | 0.96 | 0.96 | 0.94 | 0.14 | 0.86 | 0.95 |  | |
|  | H-wk3 | 0.80 | 0.75 | 0.75^z^ | 0.98^y^ | 0.16 | 0.46 | 0.26 |  | |
|  | H-wk4 | 0.89 | 0.99 | 0.67^b^ | 1.02^a^ | 0.16 | 0.09 | 0.35 |  | |
| Butyrate [%] | M | 6.75 | 6.08 | 6.51^y^ | 5.93^z^ | 0.45 | 0.07 | 0.89 | 0.49 | |
|  | H-wk1 | 7.26 | 7.45 | 7.13 | 6.37 | 0.64 | 0.58 | 0.35 |  | |
|  | H-wk2 | 7.37 | 7.42 | 7.10 | 7.43 | 0.71 | 0.74 | 0.80 |  | |
|  | H-wk3 | 7.43 | 7.46 | 7.58 | 7.17 | 0.71 | 0.73 | 0.70 |  | |
|  | H-wk4 | 6.97 | 6.11 | 8.01^a^ | 6.82^b^ | 0.67 | 0.06 | 0.76 |  | |
| Iso-Valerate [%] | M | 0.42 | 0.42 | 0.44 | 0.51 | 0.10 | 0.68 | 0.63 | 0.54 | |
|  | H-wk1 | 0.36 | 0.33 | 0.29 | 0.33 | 0.07 | 0.95 | 0.61 |  | |
|  | H-wk2 | 0.34 | 0.34 | 0.31 | 0.33 | 0.07 | 0.93 | 0.86 |  | |
|  | H-wk3 | 0.24 | 0.24 | 0.23^z^ | 0.34^y^ | 0.08 | 0.39 | 0.35 |  | |
|  | H-wk4 | 0.27 | 0.30 | 0.19^z^ | 0.29^y^ | 0.07 | 0.24 | 0.48 |  | |
| Valerate [%] | M | 2.08 | 2.01 | 1.95 | 1.94 | 0.60 | 0.93 | 0.95 | 0.92 | |
|  | H-wk1 | 1.86 | 2.27 | 1.83 | 1.69 | 0.45 | 0.70 | 0.44 |  | |
|  | H-wk2 | 1.87 | 2.15 | 1.73 | 1.68 | 0.46 | 0.74 | 0.65 |  | |
|  | H-wk3 | 1.17 | 1.16 | 1.12 | 1.29 | 0.09 | 0.63 | 0.56 |  | |
|  | H-wk4 | 1.12 | 1.12 | 1.06^z^ | 1.23^y^ | 0.07 | 0.68 | 0.15 |  | |
| Caproate [%] | M | 0.15 | 0.22 | 0.16 | 0.18 | 0.03 | 0.09 | 0.31 | 0.36 | |
|  | H-wk1 | 0.15 | 0.15 | 0.14 | 0.14 | 0.02 | 0.93 | 0.86 |  | |
|  | H-wk2 | 0.13 | 0.14 | 0.15 | 0.14 | 0.02 | 0.88 | 0.68 |  | |
|  | H-wk3 | 0.12 | 0.10 | 0.14 | 0.12 | 0.02 | 0.36 | 0.92 |  | |
|  | H-wk4 | 0.11 | 0.11 | 0.11 | 0.11 | 0.02 | 0.95 | 0.78 |  | |
| Lactate [mM] | M | 0.41 | 0.28 | 0.25 | 0.13 | 0.20 | 0.43 | 0.98 | 0.06 | |
|  | H-wk1 | 0.17^b^ | 0.53^a^ | 0.12 | 0.12 | 0.12 | 0.06 | 0.06 |  | |
|  | H-wk2 | 0.15^z^ | 0.61^y^ | 0.13 | 0.22 | 0.19 | 0.08 | 0.23 |  | |
|  | H-wk3 | 0.23 | 0.09 | 0.07 | 0.07 | 0.08 | 0.27 | 0.31 |  | |
|  | H-wk4 | 0.08 | 0.10 | 0.07 | 0.11 | 0.03 | 0.24 | 0.87 |  | |
| Fecal pH | M | 6.60 | 6.63 | 6.60 | 6.70 | 0.07 | 0.28 | 0.58 | 0.08 | |
|  | H-wk1 | 6.62 | 6.63 | 6.51 | 6.54 | 0.07 | 0.67 | 0.84 |  | |
|  | H-wk2 | 6.55 | 6.45 | 6.56 | 6.50 | 0.07 | 0.19 | 0.56 |  | |
|  | H-wk3 | 6.55 | 6.47 | 6.54 | 6.55 | 0.07 | 0.54 | 0.48 |  | |
|  | H-wk4 | 6.60 | 6.56 | 6.44^b^ | 6.62^a^ | 0.07 | 0.18 | 0.35 |  | |

^a,b^ indicate differences between CON and CM-cows within parity (p ≤ 0.05).^y,z^ indicate differences by trend between CON and CM-cows within parity (0.05 < p ≤ 0.10).^1^Feeding phases were moderate-starch feeding (M) and four weeks of high-starch feeding, i.e. H-wk1 - H-wk4.^2^Control cows, i.e. without CM supplementation.^3^Cows supplemented with CM.^4^Short-chain fatty acids.

**Table S2.**

| Item | Phase | PP | | MP | | SEM^1^ | P-value | | |
| --- | --- | --- | --- | --- | --- | --- | --- | --- | --- |
|  |  | CON | CM | CON | CM |  | Additive | Parity | Additive × Parity |
| Glucose [mg/dL] | M | 64.8 | 65.3 | 64.5 | 66.5 | 3.47 | 0.68 | 0.90 | 0.79 |
|  | H-Wk1 | 71.1 | 72.4 | 64.7 | 66.7 | 3.01 | 0.51 | 0.03 | 0.87 |
|  | H-Wk2 | 68.0 | 68.7 | 64.5 | 66.2 | 2.48 | 0.58 | 0.19 | 0.80 |
|  | H-Wk3 | 70.9 | 70.2 | 65.8 | 68.2 | 2.53 | 0.70 | 0.14 | 0.43 |
|  | H-Wk4 | 69.6 | 68.4 | 69.9 | 66.1 | 2.75 | 0.27 | 0.69 | 0.54 |
| Cholesterol [mg/dL] | M | 143 | 155 | 163 | 153 | 10.9 | 0.92 | 0.32 | 0.20 |
|  | H-Wk1 | 149 | 148 | 154 | 146 | 10.0 | 0.56 | 0.82 | 0.69 |
|  | H-Wk2 | 155 | 142 | 146 | 153 | 12.5 | 0.74 | 0.88 | 0.30 |
|  | H-Wk3 | 155 | 155 | 151 | 155 | 16.5 | 0.87 | 0.86 | 0.86 |
|  | H-Wk4 | 170 | 163 | 156 | 159 | 18.5 | 0.89 | 0.56 | 0.73 |
| BHBA^2^ [mmol/L] | M | 0.40 | 0.318 | 0.38 | 0.34 | 0.07 | 0.26 | 0.98 | 0.65 |
|  | H-Wk1 | 0.36 | 0.310 | 0.34 | 0.31 | 0.06 | 0.39 | 0.86 | 0.81 |
|  | H-Wk2 | 0.28 | 0.413 | 0.35 | 0.32 | 0.07 | 0.34 | 0.88 | 0.12 |
|  | H-Wk3 | 0.28 | 0.297 | 0.35 | 0.33 | 0.07 | 1.00 | 0.39 | 0.71 |
|  | H-Wk4 | 0.30 | 0.384 | 0.32 | 0.27 | 0.05 | 0.66 | 0.31 | 0.10 |
| NEFA^3^ [mmol/L] | M | 0.10 | 0.01 | 0.21 | 0.18 | 0.07 | 0.33 | 0.02 | 0.64 |
|  | H-Wk1 | 0.09 | 0.04 | 0.13 | 0.08 | 0.05 | 0.17 | 0.23 | 1.00 |
|  | H-Wk2 | 0.15 | 0.06 | 0.09 | 0.07 | 0.05 | 0.22 | 0.55 | 0.39 |
|  | H-Wk3 | 0.06 | 0.04 | 0.08 | 0.07 | 0.03 | 0.49 | 0.44 | 0.74 |
|  | H-Wk4 | 0.07 | 0.04 | 0.15 | 0.08 | 0.05 | 0.32 | 0.19 | 0.75 |
| Triglycerides [mg/dL] | M | 9.31^b^ | 13.1^a^ | 12.3 | 11.0 | 1.38 | 0.25 | 0.68 | 0.03 |
|  | H-Wk1 | 8.34^b^ | 12.4^a^ | 10.1 | 10.8 | 1.46 | 0.05 | 0.97 | 0.15 |
|  | H-Wk2 | 9.83 | 8.40 | 9.57 | 9.44 | 1.29 | 0.45 | 0.72 | 0.52 |
|  | H-Wk3 | 11.2 | 8.83 | 8.67 | 8.92 | 1.30 | 0.30 | 0.26 | 0.20 |
|  | H-Wk4 | 8.25 | 10.0 | 10.1 | 9.04 | 1.36 | 0.74 | 0.71 | 0.20 |
| Albumin [g/dL] | M | 4.01^b^ | 4.63^a^ | 4.02 | 4.10 | 0.19 | 0.03 | 0.09 | 0.08 |
|  | H-Wk1 | 3.76^b^ | 4.40^a^ | 3.96 | 4.14 | 0.14 | <0.01 | 0.82 | 0.08 |
|  | H-Wk2 | 3.86 | 4.13 | 3.97 | 4.17 | 0.22 | 0.18 | 0.64 | 0.84 |
|  | H-Wk3 | 4.17 | 4.36 | 3.96 | 4.36 | 0.34 | 0.28 | 0.71 | 0.69 |
|  | H-Wk4 | 4.00 | 4.25 | 3.94 | 4.23 | 0.26 | 0.19 | 0.84 | 0.89 |
| Bilirubin [mg/dL] | M | 0.06 | 0.07 | 0.08 | 0.07 | 0.01 | 0.76 | 0.33 | 0.17 |
|  | H-Wk1 | 0.06 | 0.05 | 0.10 | 0.08 | 0.02 | 0.48 | 0.02 | 0.82 |
|  | H-Wk2 | 0.08 | 0.04 | 0.08 | 0.09 | 0.03 | 0.49 | 0.16 | 0.29 |
|  | H-Wk3 | 0.07 | 0.06 | 0.08 | 0.10 | 0.03 | 0.88 | 0.22 | 0.69 |
|  | H-Wk4 | 0.06 | 0.06 | 0.10 | 0.09 | 0.02 | 0.98 | 0.04 | 0.97 |
| Calcium [mmol/L] | M | 2.43 | 2.50 | 2.61 | 2.54 | 0.09 | 0.99 | 0.21 | 0.40 |
|  | H-Wk1 | 2.39 | 2.36 | 2.49 | 2.44 | 0.11 | 0.60 | 0.35 | 0.89 |
|  | H-Wk2 | 2.71^y^ | 2.34^z^ | 2.31 | 2.46 | 0.13 | 0.35 | 0.29 | 0.04 |
|  | H-Wk3 | 2.51 | 3.55 | 2.43 | 2.51 | 0.11 | 0.97 | 0.99 | 0.35 |
|  | H-Wk4 | 2.75 | 2.52 | 2.52 | 2.45 | 0.11 | 0.15 | 0.19 | 0.43 |
| Phosphorus [mmol/L] | M | 1.57 | 1.51 | 1.55 | 1.56 | 0.19 | 0.84 | 0.91 | 0.81 |
|  | H-Wk1 | 2.29 | 2.02 | 1.72 | 1.82 | 0.19 | 0.59 | 0.03 | 0.25 |
|  | H-Wk2 | 2.13 | 2.26 | 1.90 | 1.87 | 0.27 | 0.82 | 0.18 | 0.73 |
|  | H-Wk3 | 1.92 | 1.57 | 1.91 | 2.01 | 0.20 | 0.44 | 0.21 | 0.17 |
|  | H-Wk4 | 1.98 | 2.04 | 1.66 | 1.81 | 0.18 | 0.48 | 0.08 | 0.74 |
| Magnesium [mmol/L] | M | 1.08 | 1.20 | 1.19 | 1.18 | 0.10 | 0.46 | 0.57 | 0.39 |
|  | H-Wk1 | 1.32 | 1.39 | 1.29 | 1.23 | 0.08 | 0.95 | 0.14 | 0.34 |
|  | H-Wk2 | 1.35 | 1.43 | 1.26 | 1.29 | 0.09 | 0.51 | 0.21 | 0.80 |
|  | H-Wk3 | 1.36 | 1.57 | 1.37 | 1.41 | 0.17 | 0.35 | 0.58 | 0.50 |
|  | H-Wk4 | 1.33 | 1.39 | 1.28 | 1.29 | 0.12 | 0.71 | 0.43 | 0.82 |
| AST^4^ [U/L] | M | 85.5 | 99.0 | 96.0 | 101 | 9.27 | 0.23 | 0.42 | 0.57 |
|  | H-Wk1 | 150 | 123 | 115 | 112 | 17.0 | 0.26 | 0.10 | 0.40 |
|  | H-Wk2 | 179 | 161 | 116 | 137 | 20.3 | 0.93 | 0.01 | 0.26 |
|  | H-Wk3 | 181 | 217 | 147 | 182 | 39.4 | 0.27 | 0.28 | 1.00 |
|  | H-Wk4 | 178 | 192 | 169 | 184 | 39.6 | 0.65 | 0.79 | 1.00 |
| GLDH^5^ [U/L] | M | 27.3 | 22.9 | 22.7 | 25.5 | 3.34 | 0.76 | 0.71 | 0.19 |
|  | H-Wk1 | 279^a^ | 56.3^b^ | 58.8 | 54.9 | 67.2 | 0.04 | 0.05 | 0.06 |
|  | H-Wk2 | 163 | 106 | 55.1 | 75.8 | 25.4 | 0.36 | <0.01 | 0.07 |
|  | H-Wk3 | 155 | 232 | 71.0 | 112 | 54.5 | 0.21 | 0.03 | 0.71 |
|  | H-Wk4 | 131 | 159 | 73.2 | 99.8 | 40.7 | 0.41 | 0.08 | 0.99 |
| GGT^6^ [U/L] | M | 27.4 | 26.9 | 29.4 | 29.4 | 1.92 | 0.87 | 0.17 | 0.85 |
|  | H-Wk1 | 42.5^a^ | 29.0^b^ | 30.0 | 31.3 | 4.37 | 0.08 | 0.16 | 0.04 |
|  | H-Wk2 | 62.3^a^ | 34.0^b^ | 31.6 | 36.7 | 8.23 | 0.08 | 0.04 | 0.02 |
|  | H-Wk3 | 64.7 | 49.2 | 34.3 | 42.6 | 8.87 | 0.60 | 0.02 | 0.09 |
|  | H-Wk4 | 67.4 | 60.5 | 37.0 | 47.6 | 10.7 | 0.82 | 0.02 | 0.30 |
| AP^7^ [U/L] | M | 94 | 102 | 103 | 110 | 12.7 | 0.46 | 0.41 | 0.99 |
|  | H-Wk1 | 122 | 109 | 112 | 115 | 6.58 | 0.44 | 0.80 | 0.18 |
|  | H-Wk2 | 113 | 123 | 110 | 114 | 8.40 | 0.33 | 0.38 | 0.72 |
|  | H-Wk3 | 118 | 119 | 106 | 113 | 6.12 | 0.44 | 0.08 | 0.61 |
|  | H-Wk4 | 137 | 133 | 103 | 120 | 15.89 | 0.63 | 0.08 | 0.41 |
| Haptoglobin [μg/mL] | M | 238 | 292 | 722^a^ | 111^b^ | 260.6 | 0.20 | 0.48 | 0.13 |
|  | H-Wk1 | 6198 | 1075 | 966 | 592 | 2180 | 0.19 | 0.17 | 0.25 |
|  | H-Wk2 | 275 | 283 | 109 | 166 | 125 | 0.75 | 0.18 | 0.81 |
|  | H-Wk3 | 1329 | 1018 | 1322 | 715 | 1328 | 0.67 | 0.89 | 0.89 |
|  | H-Wk4 | 206 | 215 | 367 | 273 | 192 | 0.79 | 0.49 | 0.74 |
| SAA^8^ [μg/mL] | M | 48.0 | 50.1 | 78.8 | 40.9 | 37.6 | 0.54 | 0.73 | 0.50 |
|  | H-Wk1 | 96.0 | 58.7 | 72.6 | 79.8 | 37.9 | 0.61 | 0.97 | 0.46 |
|  | H-Wk2 | 51.7 | 71.9 | 38.4 | 37.2 | 33.5 | 0.72 | 0.39 | 0.68 |
|  | H-Wk3 | 36.9 | 13.8 | 63.4 | 63.4 | 43.0 | 0.73 | 0.29 | 0.73 |
|  | H-Wk4 | 16.5 | 17.6 | 75.3 | 61.2 | 32.6 | 0.80 | 0.07 | 0.77 |

^1^SEM, Standard error of the mean; ^2^BHBA, β-hydroxybutyrate; ^3^NEFA, non-esterified fatty acids; ^4^AST, aspartate aminotransferase; ^5^GLDH, glutamate dehydrogenase; ^6^GGT, γ-glutamyl transferase; ^7^AP, alkaline phosphatase; ^8^SAA, serum amyloid A. ^a,b^ indicate differences between CON and CM within parity (p ≤ 0.05). ^y,z^ indicate differences by trend between CON and CM within parity (0.05 < p ≤ 0.10).

**TABLE S3**. Effects of clay mineral-based feed additive (CM) and interaction CM × parity on the serum metabolome during moderate-starch feeding, and the first and fourth week of high-starch feeding.

| **Metabolite (μM)** | Moderate-grain feeding | | | | SEM | P-value | |
| --- | --- | --- | --- | --- | --- | --- | --- |
|  | Primiparous | | Multiparous | |  |  |  |
|  | CON | CM | CON | CM |  | CM | CM x Parity |
| **Moderate-starch feeding** |  |  |  |  |  |  |  |
| **Amino Acids** |  |  |  |  |  |  |  |
| Glycine | 258 | 330 | 367 | 434 | 50.9 | 0.04 | 0.56 |
| Valine | 218 | 198 | 185 | 171 | 17.0 | 0.10 | 0.89 |
| **Bile Acids** |  |  |  |  |  |  |  |
| Cholic acid | 9.41 | 19.4 | 25.6^b^ | 74.0^a^ | 38.9 | 0.01 | 0.49 |
| Chenodeoxycholic acid | 0.11 | 0.35 | 0.48^z^ | 1.70^y^ | 1.49 | 0.03 | 0.87 |
| Deoxycholic acid | 0.24 | 0.70 | 1.24^z^ | 3.41^y^ | 1.86 | 0.02 | 0.91 |
| **Biogenic Amines** |  |  |  |  |  |  |  |
| Putrescine | 0.07 | 0.08 | 0.09 | 0.08 | 0.01 | 0.82 | 0.08 |
| **Amino Acid Related** |  |  |  |  |  |  |  |
| 1-Methylhistidine | 4.12 | 3.60 | 4.93^y^ | 3.96^z^ | 0.54 | 0.08 | 0.68 |
| Homocysteine | 14.8^a^ | 10.7^b^ | 14.0^y^ | 11.9^z^ | 1.19 | 0 | 0.22 |
| **Hormones and Related** |  |  |  |  |  |  |  |
| Abscisic acid | 0.01 | 0.02 | 0.01^z^ | 0.02^y^ | 0.01 | 0.02 | 0.87 |
| **Ceramides and Derivatives** |  |  |  |  |  |  |  |
| Cerd181180 | 0.09 | 0.13 | 0.14^z^ | 0.26^y^ | 0.41 | 0.01 | 0.27 |
| **Diglycerides** |  |  |  |  |  |  |  |
| DG(16:0_16:0) | 7.25^y^ | 5.84^z^ | 6.56 | 7.42 | 0.59 | 0.47 | 0.01 |
| DG(18:1_18:2) | 1.23^y^ | 0.91^z^ | 0.99 | 1.29 | 0.15 | 0.76 | 0.08 |
| DG(18:1_20:1) | 0.09 | 0.07 | 0.1 | 0.13 | 0.02 | 0.52 | 0.03 |
| **Carboxylic acids** |  |  |  |  |  |  |  |
| Succinic acid | 3.2 | 2.82 | 3.21^a^ | 2.69^b^ | 0.24 | 0.04 | 0.69 |
| **Acylcarnitines** |  |  |  |  |  |  |  |
| Butyrylcarnitine | 0.11 | 0.1 | 0.09^y^ | 0.07^z^ | 0.01 | 0.1 | 0.56 |
| Hydroxyvalerylcarnitine | 0.08 | 0.07 | 0.06 | 0.06 | 0.01 | 0.63 | 0.09 |
| Nonaylcarnitine | 0.02 | 0.03 | 0.02^y^ | 0.02^z^ | 0.00 | 0.86 | 0.04 |
| Hydroxytetradecadienoylcarnitine | 0.01 | 0.01 | 0.01 | 0.01 | 0.00 | 0.45 | 0.05 |
| **Sphingomyelins** |  |  |  |  |  |  |  |
| SM C26:1 | 0.15^z^ | 0.42^y^ | 0.33 | 0.20 | 0.05 | 0.46 | 0.04 |
| **Fatty acids** |  |  |  |  |  |  |  |
| Docosahexaenoic acid | 0.30 | 0.37 | 0.35 | 0.44 | 0.08 | 0.08 | 0.82 |
| **Phosphatidylcholines** |  |  |  |  |  |  |  |
| PC ae C34:2 | 21.5 | 25.2 | 28.4 | 25.8 | 2.19 | 0.55 | 0.08 |
| PC aa C26:0 | 2.53 | 2.37 | 3.17^a^ | 2.11^b^ | 0.23 | 0.04 | 0.19 |
| **Lysophosphatidylcholines** |  |  |  |  |  |  |  |
| lysoPC a C14:0 | 1.90 | 2.08 | 2.01^z^ | 2.17^y^ | 0.08 | 0.05 | 0.79 |
| lysoPC a C24:0 | 0.53 | 0.5 | 0.55^a^ | 0.42^b^ | 0.05 | 0.05 | 0.36 |
| **Triglycerides** |  |  |  |  |  |  |  |
| TG(16:1_36:3) | 0.25 | 0.35 | 0.40 | 0.46 | 0.07 | 0.09 | 0.54 |
| TG(18:1_30:1) | 0.78^a^ | 0.68^b^ | 0.71 | 0.47 | 0.14 | 0.07 | 0.32 |
| TG(18:1_36:5) | 0.49^y^ | 0.32^z^ | 0.47 | 0.40 | 0.07 | 0.09 | 0.28 |
| TG(18:2_32:2) | 0.30 | 0.31 | 0.29 | 0.40 | 0.17 | 0.08 | 0.59 |
| TG(18:2_33:1) | 0.34 | 0.38 | 0.44^b^ | 0.68^a^ | 0.13 | 0.06 | 0.27 |
| **First week of high-starch feeding** | | |  |  |  |  |  |
| **Amino Acids** |  |  |  |  |  |  |  |
| Glutamate | 64.4^a^ | 43.4^b^ | 44.3 | 46.7 | 4.52 | 0.04 | 0.00 |
| Histidine | 45.5 | 56.4 | 62.3 | 67.0 | 9.45 | 0.09 | 0.24 |
| **Bile Acids** |  |  |  |  |  |  |  |
| Glycolithocolic acid | 0.08^b^ | 0.18^a^ | 0.15 | 0.13 | 0.04 | 0.15 | 0.05 |
| **Biogenic Amines** |  |  |  |  |  |  |  |
| Histamine | 0.24^z^ | 0.24^y^ | 0.24 | 0.24 | 0.00 | 0.10 | 0.29 |
| **Hormones and Related** |  |  |  |  |  |  |  |
| Abscisic acid | 0.01 | 0.03 | 0.02^y^ | 0.01^z^ | 0.01 | 0.78 | 0.06 |
| **Ceramides and Derivatives** |  |  |  |  |  |  |  |
| CE(15:1) | 2.07 | 2.6 | 2.37 | 2.29 | 0.27 | 0.11 | 0.03 |
| Hex2Cer(d18:1/16:0) | 0.67 | 0.83 | 0.81 | 0.73 | 0.07 | 0.58 | 0.04 |
| Hex2Cer(d18:1/24:0) | 0.13 | 0.1 | 0.11 | 0.09 | 0.04 | 0.02 | 0.25 |
| Hex3Cer(d18:1/16:0) | 0.63 | 0.73 | 0.76 | 0.66 | 0.09 | 1.00 | 0.09 |
| **Acylcarnitines** |  |  |  |  |  |  |  |
| Butyrylcarnitine | 0.13^a^ | 0.10^b^ | 0.09 | 0.08 | 0.01 | 0.02 | 0.1 |
| Glutarylcarnitine | 0.03^b^ | 0.11^a^ | 0.05^b^ | 0.07^a^ | 0.02 | 0.00 | 0.16 |
| Methylglutarylcarnitine | 0.03 | 0.02 | 0.02 | 0.03 | 0.00 | 0.94 | 0.09 |
| Pimeloylcarnitine | 0.02 | 0.02 | 0.01^z^ | 0.03^y^ | 0.01 | 0.78 | 0.07 |
| Dodecenoylcarnitine | 0.04 | 0.04 | 0.03^b^ | 0.04^a^ | 0.02 | 0.15 | 0.09 |
| Tetradecadienoylcarnitine | 0.03^z^ | 0.04^y^ | 0.04 | 0.04 | 0.00 | 0.52 | 0.02 |
| Hexadecanoylcarnitine | 0.03 | 0.03 | 0.03 | 0.03 | 0.00 | 0.77 | 0.08 |
| Hexadecenoylcarnitine | 0.02 | 0.02 | 0.02 | 0.02 | 0.00 | 0.07 | 0.39 |
| **Sphingomyelins** |  |  |  |  |  |  |  |
| SM (OH) C22:1 | 10.7 | 12.3 | 13.8 | 12.9 | 1.08 | 0.46 | 0.10 |
| **Fatty acids** |  |  |  |  |  |  |  |
| Eicosadienoic acid | 0.34^y^ | 0.24^z^ | 0.32 | 0.48 | 0.10 | 0.84 | 0.07 |
| **Phosphatidylcholines** |  |  |  |  |  |  |  |
| PC aa C30:0 | 2.72^z^ | 3.26^y^ | 3.40 | 3.51 | 0.25 | 0.07 | 0.23 |
| PC aa C36:0 | 5.65 | 6.96 | 6.23 | 6.71 | 0.57 | 0.07 | 0.43 |
| PC aa C40:2 | 0.46 | 0.50 | 0.51 | 0.63 | 0.06 | 0.06 | 0.47 |
| PC aa C42:0 | 0.13b | 0.2a | 0.17 | 0.17 | 0.02 | 0.08 | 0.09 |
| **Lysophosphatidylcholines** |  |  |  |  |  |  |  |
| lysoPC a C16:0 | 8.46 | 9.27 | 10.8^a^ | 9.23^b^ | 0.70 | 0.55 | 0.02 |
| lysoPC a C16:1 | 0.66 | 0.75 | 0.93^a^ | 0.74^b^ | 0.07 | 0.44 | 0.01 |
| lysoPC a C18:2 | 12.0^z^ | 15.3^y^ | 18.4^y^ | 15.7^z^ | 1.70 | 0.62 | 0.02 |
| lysoPC a C20:3 | 1.86^z^ | 2.56^y^ | 2.5 | 2.43 | 0.29 | 0.11 | 0.07 |
| lysoPC a C20:4 | 1.19 | 1.46 | 1.42 | 1.27 | 0.17 | 0.65 | 0.09 |
| **Triglycerides** |  |  |  |  |  |  |  |
| TG(14:0_34:2) | 0.52 | 0.62 | 0.49 | 0.58 | 0.07 | 0.07 | 0.93 |
| TG(14:0_35:1) | 0.31^z^ | 0.55^y^ | 0.43 | 0.30 | 0.09 | 0.51 | 0.06 |
| TG(14:0_36:3) | 0.46 | 0.38 | 0.37 | 0.5 | 0.12 | 0.80 | 0.10 |
| TG(16:0_28:1) | 0.8 | 0.44 | 0.44 | 0.47 | 0.15 | 0.20 | 0.09 |
| TG(16:0_35:2) | 0.6 | 0.74 | 0.46^z^ | 0.60^y^ | 0.09 | 0.04 | 0.65 |
| TG(16:0_36:2) | 3.28 | 3.91 | 2.94^z^ | 3.81^y^ | 0.45 | 0.08 | 0.89 |
| TG(16:1_34:1) | 1.04 | 1.23 | 1.01 | 1.20 | 0.15 | 0.03 | 0.74 |
| TG(16:1_34:2) | 0.59^b^ | 0.98^a^ | 0.69 | 0.7 | 0.09 | 0.02 | 0.05 |
| TG(16:1_36:1) | 0.44^b^ | 0.85^a^ | 0.57 | 0.64 | 0.11 | 0.01 | 0.03 |
| TG(16:1_36:3) | 0.39 | 0.53 | 0.37 | 0.49 | 0.07 | 0.09 | 0.59 |
| TG(17:0_34:1) | 0.87 | 1.11 | 0.74 | 0.9 | 0.16 | 0.08 | 0.65 |
| TG(18:0_34:3) | 0.52 | 0.69 | 0.44 | 0.52 | 0.10 | 0.05 | 0.56 |
| TG(18:0_36:2) | 2.08^z^ | 2.89^y^ | 2.10 | 2.26 | 0.36 | 0.10 | 0.22 |
| TG(18:0_36:4) | 0.9 | 1.01 | 0.86^z^ | 1.03^y^ | 0.09 | 0.06 | 0.64 |
| TG(18:1_32:2) | 0.53 | 0.66 | 0.54^b^ | 0.82^a^ | 0.11 | 0.03 | 0.59 |
| TG(18:1_36:5) | 0.37 | 0.47 | 0.39^b^ | 0.59^a^ | 0.07 | 0.04 | 0.28 |
| TG(18:2_32:1) | 0.64 | 0.79 | 0.63^b^ | 0.84^a^ | 0.09 | 0.03 | 0.73 |
| TG(18:2_36:0) | 1.21z | 1.73y | 1.21 | 1.38 | 0.21 | 0.03 | 0.33 |
| TG(18:3_36:2) | 3.87 | 3.78 | 3.63^b^ | 4.49^a^ | 0.36 | 0.06 | 0.48 |
| TG(18:3_36:3) | 6.49 | 6.65 | 6.88 | 7.34 | 0.39 | 0.55 | 0.10 |
| TG(20:1_24:3) | 0.28^y^ | 0.18^z^ | 0.24 | 0.28 | 0.11 | 0.31 | 0.05 |
| **Fourth week of high-starch feeding** | | |  |  |  |  |  |
| **Biogenic Amines** |  |  |  |  |  |  |  |
| Spermine | 0.13 | 0.31 | 0.16 | 0.22 | 0.20 | 0.04 | 0.53 |
| **Indoles and Derivatives** |  |  |  |  |  |  |  |
| 3-Indolepropionic acid | 0.18 | 0.12 | 0.21^a^ | 0.14^b^ | 0.05 | 0.04 | 0.74 |
| **Amino Acid Related** |  |  |  |  |  |  |  |
| 5-Aminovaleric acid | 0.94^z^ | 1.88^y^ | 1.61 | 1.94 | 0.92 | 0.03 | 0.20 |
| α-Aminobutyric acid | 3.71 | 4.06 | 4.66^a^ | 2.80^b^ | 0.77 | 0.12 | 0.03 |
| Betaine | 10.6 | 7.62 | 22.2^a^ | 13.4^b^ | 5.19 | 0.06 | 0.77 |
| Homocysteine | 15.9^a^ | 11.3^b^ | 13.2 | 14.1 | 1.19 | 0.12 | 0.03 |
| Methionine sulfoxide | 1.59 | 1.12 | 1.21 | 1.09 | 0.21 | 0.09 | 0.49 |
| Symmetric dimethylarginine | 0.48 | 0.57 | 0.46 | 0.56 | 0.06 | 0.05 | 0.85 |
| Taurine | 52.3^z^ | 72.5^y^ | 65.7 | 71.9 | 6.21 | 0.01 | 0.13 |
| **Cresols** |  |  |  |  |  |  |  |
| p-Cresol sulfate | 50.3 | 61.9 | 42.6 | 55.1 | 8.11 | 0.05 | 0.90 |
| **Hormones and Related** |  |  |  |  |  |  |  |
| Cortisol | 0.01^b^ | 0.04^a^ | 0.03^b^ | 0.05^a^ | 0.03 | 0.03 | 0.19 |
| Dehydroepiandrosterone sulfate | 1.44 | 1.55 | 1.55 | 1.57 | 0.06 | 0.09 | 0.21 |
| **Ceramides and Derivatives** |  |  |  |  |  |  |  |
| CE(15:1) | 2.00^b^ | 3.14^a^ | 2.28 | 2.86 | 0.27 | 0.01 | 0.30 |
| Cer(d18:1/24:0) | 0.28 | 0.35 | 0.32 | 0.29 | 0.03 | 0.44 | 0.07 |
| Cer(d18:1/25:0) | 0.11^b^ | 0.24^a^ | 0.18 | 0.14 | 0.04 | 0.04 | 0.00 |
| Hex2Cer(d18:1/18:0) | 0.10 | 0.14 | 0.13 | 0.11 | 0.02 | 0.43 | 0.07 |
| **Acylcarnitines** |  |  |  |  |  |  |  |
| Glutarylcarnitine | 0.08 | 0.07 | 0.05 | 0.07 | 0.02 | 0.61 | 0.05 |
| Hydroxyvalerylcarnitine | 0.08 | 0.06 | 0.06^z^ | 0.07^y^ | 0.01 | 0.90 | 0.10 |
| Dodecanedioylcarnitine | 0.39 | 0.36 | 0.39 | 0.43 | 0.02 | 0.87 | 0.04 |
| Hydroxytetradecadienoylcarnitine | 0.02^y^ | 0.01^z^ | 0.01^z^ | 0.02^y^ | 0.00 | 0.87 | 0.04 |
| **Sphingomyelins** |  |  |  |  |  |  |  |
| SM C26:1 | 0.10^b^ | 0.38^a^ | 0.16 | 0.21 | 0.05 | 0.07 | 0.33 |
| **Fatty acids** |  |  |  |  |  |  |  |
| Arachidonic acid | 0.82 | 1.19 | 0.95 | 0.84 | 0.15 | 0.41 | 0.09 |
| Eicosapentaenoic acid | 0.77 | 1.10 | 0.97 | 0.82 | 0.20 | 0.50 | 0.07 |
| **Phosphatidylcholines** |  |  |  |  |  |  |  |
| PC ae C30:2 | 0.56^b^ | 0.76^a^ | 0.6 | 0.69 | 0.06 | 0.02 | 0.25 |
| PC ae C42:3 | 0.39^z^ | 0.49^y^ | 0.42 | 0.42 | 0.04 | 0.10 | 0.09 |
| PC ae C44:5 | 0.15 | 0.19 | 0.15 | 0.16 | 0.02 | 0.07 | 0.17 |
| PC aa C30:2 | 0.27 | 0.34 | 0.24^z^ | 0.29^y^ | 0.03 | 0.03 | 0.96 |
| PC aa C38:0 | 3.76 | 3.04 | 3.15^a^ | 2.52^b^ | 0.35 | 0.03 | 0.99 |
| PC aa C40:3 | 6.80 | 5.74 | 8.66^y^ | 6.90^z^ | 1.17 | 0.08 | 0.83 |
| PC aa C42:5 | 1.15 | 0.97 | 1.07 | 0.85 | 0.15 | 0.03 | 0.87 |
| **Lysophosphatidylcholines** |  |  |  |  |  |  |  |
| lysoPC a C14:0 | 2.02 | 2.14 | 2.13 | 2.19 | 0.08 | 0.08 | 0.53 |
| lysoPC a C28:1 | 1.88^z^ | 2.45^y^ | 2.12 | 2.5 | 0.28 | 0.03 | 0.50 |
| **Triglycerides** |  |  |  |  |  |  |  |
| TG(14:0_35:1) | 0.38 | 0.49 | 0.33 | 0.38 | 0.09 | 0.10 | 0.95 |
| TG(14:0_36:2) | 0.52 | 0.73 | 0.6 | 0.68 | 0.12 | 0.04 | 0.53 |
| TG(16:0_36:4) | 1.61 | 1.77 | 1.67 | 1.82 | 0.11 | 0.09 | 0.92 |
| TG(17:0_32:1) | 0.59 | 0.69 | 0.54^z^ | 0.71^y^ | 0.08 | 0.02 | 0.41 |
| TG(18:1_26:0) | 0.55^b^ | 0.91^a^ | 0.57 | 0.51 | 0.14 | 0.29 | 0.09 |
| TG(18:1_35:2) | 0.33^b^ | 0.57^a^ | 0.48 | 0.48 | 0.66 | 0.01 | 0.01 |
| TG(18:2_30:1) | 0.27 | 0.41 | 0.32 | 0.42 | 0.09 | 0.04 | 0.65 |
| TG(18:2_32:2) | 0.35 | 0.19 | 0.37 | 0.48 | 0.17 | 0.80 | 0.01 |

^a,b^ indicate differences between CON and CM-cows within parity (p ≤ 0.05).

^y,z^ indicate differences by trend between CON and CM-cows within parity (0.05 < p ≤ 0.10).

**TABLE S4**. Effects of clay mineral-based feed additive on dry matter intake (DMI), and mean rumen pH during moderate-starch feeding, and four weeks of high-starch feeding.

|  |  |  | **Phase^1^** | | | | |  |  |
| --- | --- | --- | --- | --- | --- | --- | --- | --- | --- |
| **Item** |  |  | **M** | **H-wk1** | **H-wk2** | **H-wk3** | **H-wk4** | **SEM** | **P-value** |
| DMI [kg/d] | CON^2^ |  | 20.6 | 20.3 | 22.6 | 22.5 | 22.7 | 1.53 | 0.99 |
|  | CM^3^ |  | 20.4 | 21.0 | 22.8 | 22.3 | 22.6 |  |  |
| Milk yield [kg/d] | CON |  | 38.0 | 39.6 | 41.1 | 41.9 | 41.3 | 2.43 | 0.31 |
|  | CM |  | 39.5 | 41.6 | 43.2 | 43.0 | 43.3 |  |  |
| SARA index^4^ | CON |  | 13.7 | 16.1 | 11.8 | 12.8 | 13.0 | 4.22 | 0.60 |
|  | CM |  | 13.5 | 14.3 | 9.62 | 10.1 | 10.8 |  |  |

^1^Feeding phases were moderate-starch feeding (M) and four weeks of high-starch feeding, i.e. H-wk1 - H-wk4.^2^Control cows, i.e. without CM supplementation.^3^Cows supplemented with CM. 4The SARA (sub-acute rumen acidosis) index was calculated as the area of ruminal pH below 6.0 (6 - pH × min), normalized for DMI.
